# Proteotoxicity in cardiac amyloidosis: amyloidogenic light chains affect the levels of intracellular proteins in human heart cells

**DOI:** 10.1038/s41598-017-15424-3

**Published:** 2017-11-15

**Authors:** Esther Imperlini, Massimiliano Gnecchi, Paola Rognoni, Eduard Sabidò, Maria Chiara Ciuffreda, Giovanni Palladini, Guadalupe Espadas, Francesco Mattia Mancuso, Margherita Bozzola, Giuseppe Malpasso, Veronica Valentini, Giuseppina Palladini, Stefania Orrù, Giovanni Ferraro, Paolo Milani, Stefano Perlini, Francesco Salvatore, Giampaolo Merlini, Francesca Lavatelli

**Affiliations:** 1 IRCCS SDN, Naples, Italy; 2 CEINGE–Biotecnologie Avanzate, Naples, Italy; 3 Coronary Care Unit and Laboratory of Experimental Cardiology for Cell and Molecular Therapy, Fondazione IRCCS Policlinico San Matteo, Pavia, Italy; 4 Department of Molecular Medicine, Unit of Cardiology, University of Pavia, Pavia, Italy; 5 Department of Medicine, University of Cape Town, Cape Town, South Africa; 6 Amyloidosis Research and Treatment Center, Department of Molecular Medicine, Fondazione IRCCS Policlinico San Matteo and University of Pavia, Pavia, Italy; 7 Centre for Genomic Regulation (CRG), The Barcelona Institute for Science and Technology, Barcelona, Spain; 8 Universitat Pompeu Fabra (UPF), Barcelona, Spain; 9 Department of Internal Medicine, Fondazione IRCCS Policlinico San Matteo and University of Pavia, Pavia, Italy; 10 Department of Movement Sciences, “Parthenope” University, Naples, Italy; 11 Department of Molecular Medicine and Medical Biotechnologies, University of Naples “Federico II”, Pavia, Italy

## Abstract

AL amyloidosis is characterized by widespread deposition of immunoglobulin light chains (LCs) as amyloid fibrils. Cardiac involvement is frequent and leads to life-threatening cardiomyopathy. Besides the tissue alteration caused by fibrils, clinical and experimental evidence indicates that cardiac damage is also caused by proteotoxicity of prefibrillar amyloidogenic species. As in other amyloidoses, the damage mechanisms at cellular level are complex and largely undefined. We have characterized the molecular changes in primary human cardiac fibroblasts (hCFs) exposed *in vitro* to soluble amyloidogenic cardiotoxic LCs from AL cardiomyopathy patients. To evaluate proteome alterations caused by a representative cardiotropic LC, we combined gel-based with label-free shotgun analysis and performed bioinformatics and data validation studies. To assess the generalizability of our results we explored the effects of multiple LCs on hCF viability and on levels of a subset of cellular proteins. Our results indicate that exposure of hCFs to cardiotropic LCs translates into proteome remodeling, associated with apoptosis activation and oxidative stress. The proteome alterations affect proteins involved in cytoskeletal organization, protein synthesis and quality control, mitochondrial activity and metabolism, signal transduction and molecular trafficking. These results support and expand the concept that soluble amyloidogenic cardiotropic LCs exert toxic effects on cardiac cells.

## Introduction

Amyloidoses are protein misfolding diseases defined by the presence of extracellular protein aggregates as cross-β-sheet amyloid fibrils. The ability to form amyloid fibrils *in vivo* is a common feature of at least 36 distinct human proteins, which are otherwise different in terms of sequence, native structure, function and localization^1^. Light chain amyloidosis (AL amyloidosis) is the most frequent systemic form, and is characterized by widespread fibril deposition in target tissues^2,3^. Causal agents of this form are misfolding-prone immunoglobulin free light chains (LCs), secreted in molar excess compared to heavy chains by a bone marrow plasma cell clone, and transported to target tissues through blood. As in the other amyloidoses, fibril deposition is associated with dysfunction of affected organs and the clinical phenotype depends on which sites are involved. In AL amyloidosis, organ involvement at presentation is heterogeneous, but cardiac deposition is present in the majority (approximately 75%) of cases, and the presence of amyloid cardiomyopathy determines survival^3^. Therefore, studying the mechanisms of heart damage and of LC cardiac tropism is of utmost importance. Although myocardial amyloid infiltration, with consequent alteration of the mechanical and structural properties of the tissue, has long been believed to be the principal culprit of the clinical manifestations, there is also evidence that soluble pre-fibrillar amyloidogenic LCs are themselves toxic for cardiac cells and that the final organ damage results from the combination of these two factors, namely, amyloid burden and direct damage by LCs^3,4^. Proteotoxicity of the soluble amyloidogenic precursors is suggested by solid clinical evidence, thanks to the availability of organ dysfunction biomarkers such as N-terminal pro-natriuretic peptide type B (NT-proBNP), BNP and troponins^5,6^. These biomarkers reflect the presence and extent of heart dysfunction and are invaluable tools with which to assess damage *in vivo*. In AL cardiomyopathy patients, in fact, variations in circulating amyloidogenic free LCs translate into parallel, concomitant and rapid changes in cardiac biomarkers, detectable after few cycles of therapy^7,8^.

The development of experimental systems that reproduce amyloidogenic light chain (LC)-mediated cardiotoxicity was an important premise to define better the molecular bases of damage caused by soluble, pre-fibrillar amyloidogenic species^9–16^. The systems used in this setting include cultured human and rodent cardiac cells^9–13,15–17^ and the recently established animal models C. *elegans* and zebrafish^11,14^. These systems share a crucial feature, namely, the fact that damage is exerted specifically by LCs that are cardiotropic in patients, and not by those that target other organs or by non-amyloidogenic LCs. In fact, exposure to exogenous cardiotropic LCs at concentrations commonly observed in patients’ sera leads to functional and cellular dysfunction. Animal cardiac cells, in particular, display a range of alterations that include impaired viability, increased reactive oxygen species (ROS) production, dysfunction and morphological damage of mitochondria^9,10,12–14^. In addition, we have demonstrated that human cells, especially cardiac fibroblasts, internalize LCs that can localize to mitochondria and interact with specific mitochondrial proteins^17^. Notably, not only amyloid deposition, but also LC proteotoxicity was shown to possess specific organ tropism. In fact, cardiotoxic LCs interact with mitochondria exclusively in cardiac fibroblasts and not in dermal ones, thereby indicating that the cell target is organ-specific^17^. However the complete landscape of molecular events occurring in target human cells has not yet been entirely explored. There is evidence that interference with cellular mechanisms contributes to LC-induced dysfunction. Therefore, the rationale of the present study is that altered protein expression profiles may be associated with the proteotoxicity of LCs, and that the description of such changes would cast light on the molecular events associated to soluble LC-induced damage.

The aim of the present work was to evaluate changes in protein abundance/representation that occur upon exposure of primary human cardiac cells (cardiac fibroblasts, hCFs) to soluble amyloidogenic cardiotropic LCs. All proteome changes were investigated using a representative pathogenic LC. To obtain maximum coverage of the proteome changes and increase the confidence of findings, we combined two independent proteomic methods: two-dimensional differential in-gel electrophoresis (2D DIGE) and label-free shotgun analysis. As a corollary to this analysis, we assessed the physiological effects of various cardiotropic and control LCs from different patients on hCFs by evaluating cell viability, cytotoxicity and injury. To verify the generalizability of the results, we explored the levels of a subset of proteins from hCFs exposed to these LCs.

Our data indicate that the amyloidogenic cardiotropic LC leads to significant remodeling of the cellular proteome, with alterations in proteins involved in key cellular processes, including cytoskeletal organization, protein synthesis and quality control, mitochondrial activity and metabolism, signal transduction and molecular trafficking. The proteome alterations are associated with changes in the cells’ physiology. The exposure to exogenous cardiotropic impairs the viability of hCFs, due to increased apoptosis, and causes oxidative stress. This investigation further supports the notion that exposure to soluble human cardiotropic LCs alters the molecular phenotype of heart cells, and points to potential mechanisms at the basis of the damage, as well as to novel potential biomarkers of LC cardiotoxicity.

## Results

**Figure 1:**
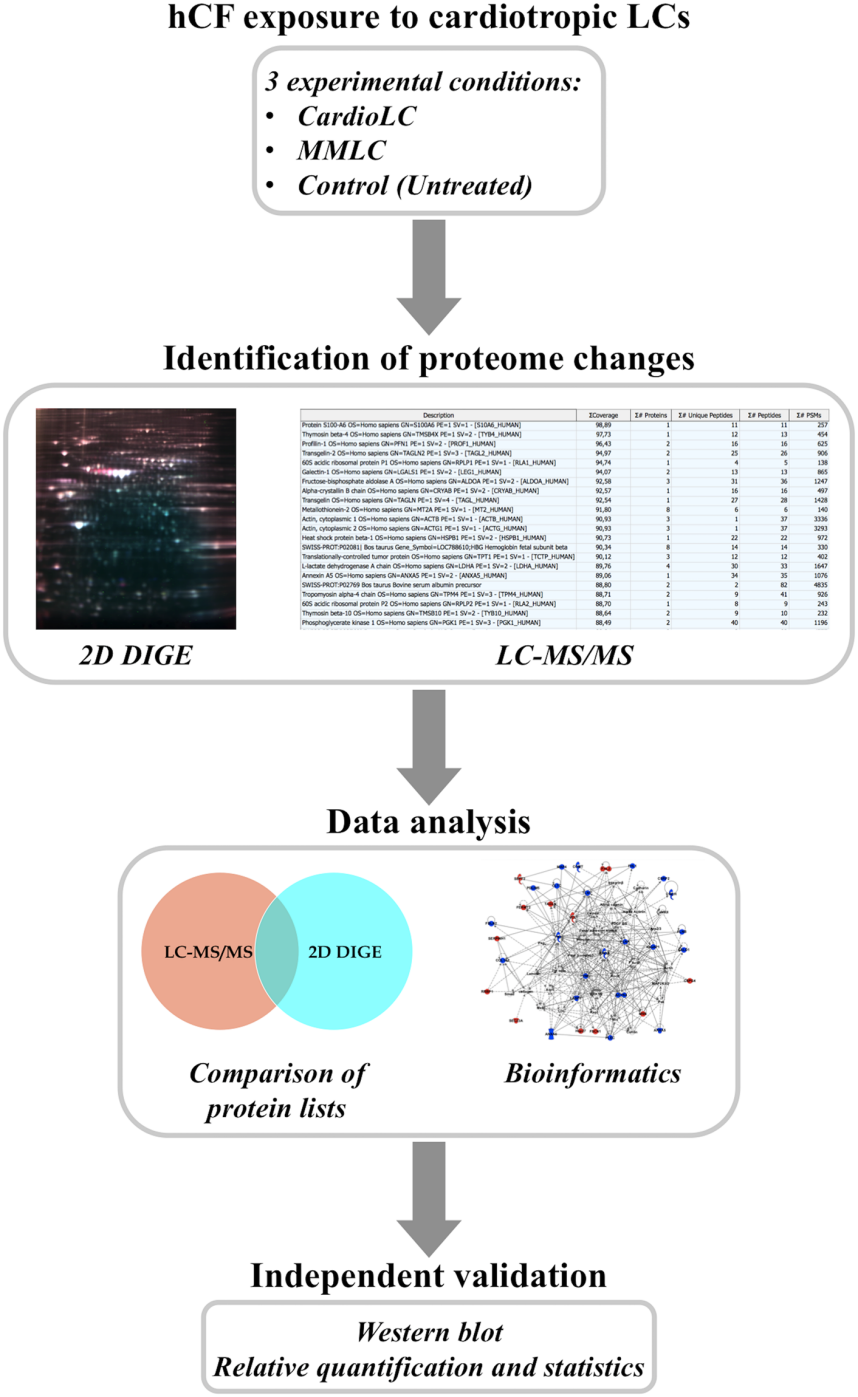
Outline of the experimental workflow. The proteome of human cardiac fibroblasts (hCFs) exposed *in vitro* to soluble cardiotoxic LCs (CardioLC) was compared with that of cells exposed to non amyloidogenic non cardiotoxic LCs from a patient with multiple myeloma (MMLC) and with untreated cells (Control). Differentially abundant proteins were identified using a combination of gel-based (2D DIGE) and gel-free (LC-MS/MS) proteomics analyses. The proteomic data were analyzed by bioinformatics and verified by western blotting.

We used a combination of proteomic and cellular assays to evaluate the molecular consequences of the exposure of hCFs to exogenous amyloidogenic cardiotoxic light chains. The clinical and biochemical features of the LCs used throughout the study are detailed in Table 1. In the proteomic assays, three experimental conditions were compared: hCFs exposed to a monoclonal amyloidogenic cardiotoxic light chain (CardioLC-1 in Table 1), hCFs exposed to a control (non amyloidogenic and non cardiotoxic) monoclonal LC from a patient with multiple myeloma (MMLC-2 in Table 1), and hCFs not exposed to LCs (Control). To identify changes in the proteome, we used two complementary techniques, 2D DIGE and LC-MS/MS-based analyses; the experimental scheme is summarized in Fig. 1.

**Table 1:** Main clinical and biochemical features of the light chains described in this study.

	LC #	GenBank #	Germline	Organs involved	Serum λ FLC (mg/l)	κ/λ FLC ratio	dFLC (mg/l)	Creati-nine (mg/dl)	Cardiac stage°	NT-proBNP (ng/l)BNP** (ng/L)	cTnI (ng/ml)	IVS mm	PW mm	EF %
Amyloidogenic (CardioLCs)	1 *	KC433671	1b(IGLV 1-51)	H	477	0.01	469	0.98	III	8882	0.16	19	19	45
	**2**	KY471436	6a (IGLV 6-57)	H, PNS, ST	839	0.002	837.5	0.74	III	1444	0.222	16.8	16	70
	**3**	KY471437	2a2(IGLV 2-14)	H, K	383	0.05	363.6	2.73	III	1926**	1.188	14.9	14.4	61
	**4**	KY471434	3 l(IGLV 3–19)	H, ST, PNS	509	0.01	500.4	0.97	III	3839	0.345	21.5	18	61
Non amyloidogenic (MMLCs)	**1**	KY471441	2b2(IGLV 2–23)	—	1140	0.001	1138.5	0.89	n.a.	201	n.a.	11	11	65
	2*	KY471438	3 l(IGLV 3–19)	—	6130	0.001	6124	2.07	n.a.	42**	0.007	9	9	65
	**3**	n.a.	2b2(IGLV 2–23)	—	573	0.011	567	0.84	n.a.	14.5	0.003	10	10.5	67

*The pair of light chains used for the proteomic analyses

Abbreviations and symbols: M, Male; F, Female; FLC, Free Light Chains; NT-proBNP, N-terminal fragment of B-type Natriuretic Peptide; cTnI, cardiac Troponin I; IVS, Interventricular Septum; PW, Posterior Wall; EF, Ejection Fraction; MW Molecular Weight; pI: Isoelectric Point.

°According to Gertz *et al*. Am J Hematol. 2005;79:319–328.

Reference ranges: serum λ FLC <26.3 mg/l, κ/λ FLC ratio 0.26–1.65; serum creatinine <1.18 mg/dl in men, <1.02 mg/dl in women; NT-proBNP <88 ng/l in men <50 years of age, <153 ng/l in women <50 years, <227 in men >50 years, <334 in women >50 years; BNP, <50 ng/l; cTnI <0.04 ng/ml.

### Identification of differentially represented proteins by 2D DIGE and by LC-MS/MS

**Figure 2:**
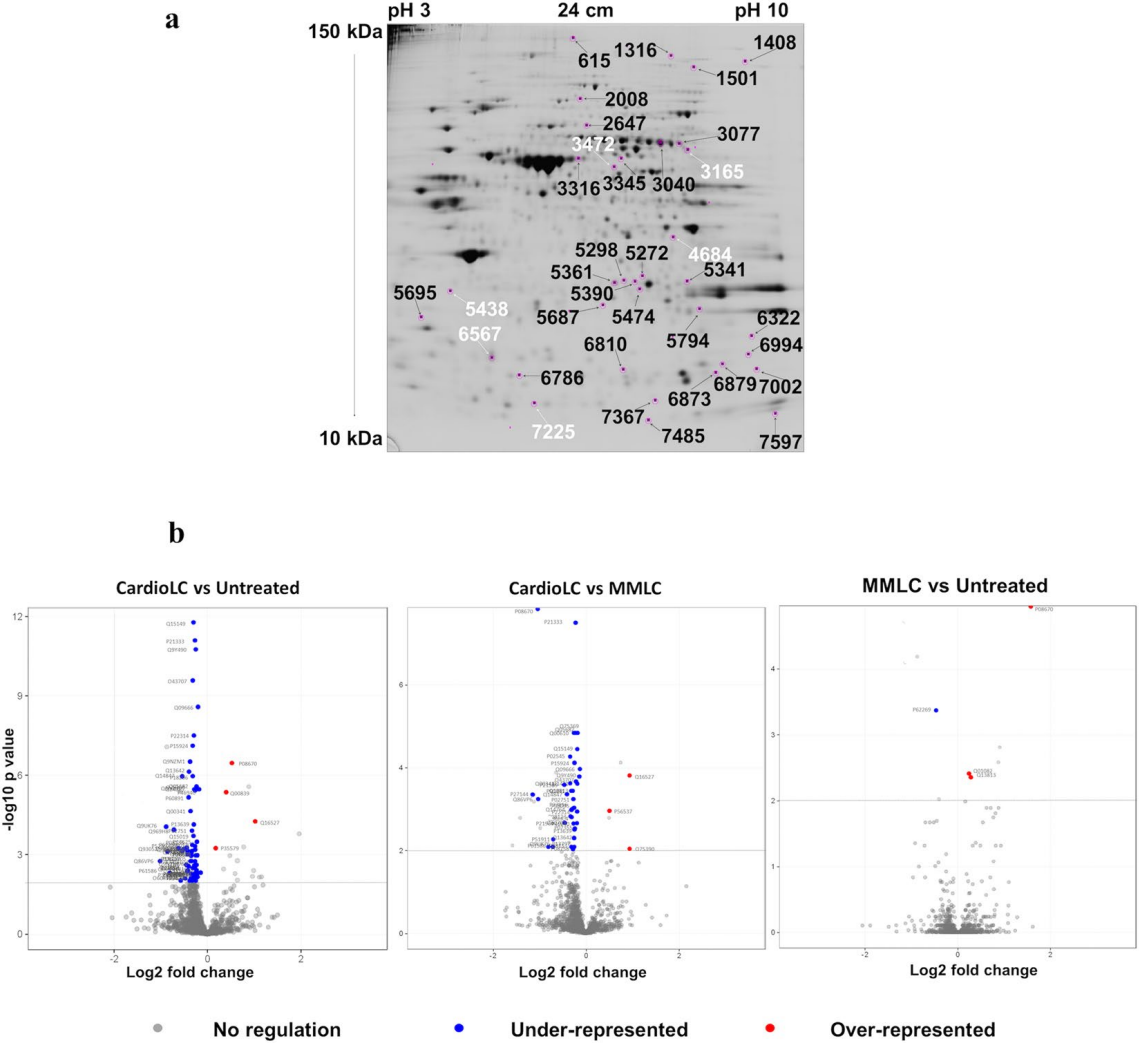
Analysis of differentially represented proteins. (**a**) Two-dimensional differential in-gel electrophoresis (2D DIGE) of hCFs exposed to cardiotoxic LCs was performed using four biological replicates. White and black labels indicate over-represented and under-represented species, respectively, identified in CardioLC-exposed hCFs compared to control or MMLC-exposed cells. (**b**) Volcano plots of the proteins identified by label-free shotgun analysis in each of the three pairwise comparisons. Three biological replicates per condition were analyzed, each one in three technical replicates. The proteins described in the manuscript (i.e. those with an adjusted *p* value < 0.01, identified with >2 distinct peptides and present in >75% replicates) are shown as colored dots and are indicated with their Uniprot accession number. Blue dots indicate under-represented proteins; red dots indicate over-represented proteins.

Quantitative 2D DIGE gel image analysis was performed to identify statistically significant (*p* value ≤ 0.05) differentially expressed protein spots with fold changes ≥ 1.20 or ≤ −1.20. Figure 2a shows a representative analytical 2D gel in which black and white numbers indicate, respectively, under- and over-represented identified spots in the CardioLC-exposed hCFs compared to control or to the MMLC-exposed cells. Overall, 40 spots were unequivocally identified as single protein species by mass spectrometry (Table 2). Indeed, to assign the observed variation to a specific protein, we considered only spots from which single species were identified, whereas spots containing two or more protein species that co-migrated in the 2D gel were excluded. The details of the identified differential protein spots, together with their cellular location, are reported in Table 2. In summary, 14 differentially represented proteins were identified in the CardioLC-exposed hCFs *versus* control cells (all under-represented) and 13 in the CardioLC *versus* MMLC-exposed hCFs comparison (10 under-represented and 3 over-represented). Two of these differential proteins, proteasome subunit beta type-2 isoform 1 (PSMB2) and ras suppressor protein 1 isoform 1 (RSU1), were shared by both comparisons with similar fold changes. LIM and SH3 domain protein 1 isoform a (LASP1) and microtubule-associated protein 1B (MAP1B) (spots 5687 and 6322, respectively) were under-represented, compared to control cells, both in CardioLC- and in MMLC-exposed hCFs. The differential proteins in hCFs exposed to the cardiotoxic LCs were localized in multiple subcellular compartments, namely cytoplasm/cytoskeleton, ribosome, endoplasmic reticulum and mitochondrion (Voltage-dependent anion-selective channel protein 1[VDAC1] or Porin 31HM).

**Table 2:** 2D DIGE analysis.

**Cardio LC** ***versus*** **control (n = 14)**								
**Spot**	**Fold** ^**a**^	***p*** **value**	**Gene**	**Protein**	**Accession**	**pI** ^**b**^	**MW** ^**b**^	**Localization**
7485	−2.41	0.042	PSMB2	proteasome subunit beta type-2 isoform 1	P49721	6.51	22993	CY, N
7367	−2.30	0.038	VDAC1	Porin 31HM	P21796	8.63	30737	M
5687	−2.19	0.049	LASP1	LIM and SH3 domain protein 1 isoform a	Q14847	6.61	30097	CS
615	−1.99	0.065	TLN1	Talin-1	Q9Y490	5.77	271653	CS
5794	−1.98	0.05	LDHA	L-lactate dehydrogenase A chain isoform 1	P00338	8.44	36950	CY
6810	−1.91	0.048	HSPB1	heat shock protein 27	P04792	7.83	22427	CY
7597	−1.67	0.044	TAGLN	transgelin	Q01995	8.87	22653	CY
6322	−1.63	0.028	MAP1B	microtubule-associated protein 1B	P46821	4.73	271651	CS
6994	−1.56	0.039	PMSA7	proteasome subunit HSPC	O14818	8.6	28057	CY, N
6879	−1.56	0.039	RSU1	ras suppressor protein 1 isoform 1	Q15404	8.57	31521	CY
7002	−1.54	0.048	RPS8	40 S ribosomal protein S8	P62241	10.32	24475	R
5695	−1.42	0.039	MAPRE1	microtubule-associated protein RP/EB family member 1	Q15691	5.02	30151	CS
1501	−1.33	0.034	LARS	leucyl tRNA synthetase	Q9P0T1	6.82	135522	CY
2008	−1.31	0.028	GANAB	glucosidase II	Q14697	5.71	107158	ER
**Cardio LC** ***versus*** **MMLC (n = 13)**								
**Spot**	**Fold** ^**a**^	***p*** **value**	**Gene**	**Protein**	**Accession**	**pI** ^**b**^	**MW** ^**b**^	**Localization**
3077	−2.86	0.05	LMNA	lamin isoform A	P02545	6.57	74380	N
3316	−2.8	0.043	DPYSL2	dihydropyrimidinase-related protein 2 isoform 2	Q16555	5.95	62711	CY, CS
7485	−2.66	0.049	PSMB2	proteasome subunit beta type-2 isoform 1	P49721	6.51	22993	CY, N
3040	−2.5	0.047	NSF	vesicle-fusing ATPase	P46459	6.52	83055	CY
3345	−1.67	0.009	CALD1	caldesmon isoform 2	Q05682	6.18	62683	CS
1408	−1.51	0.046	LDHA	L-lactate dehydrogenase A chain isoform 1	P00338	8.44	36950	CY
6879	−1.33	0.043	RSU1	ras suppressor protein 1 isoform 1	Q15404	8.57	31521	CY
843	−1.26	0.023	EEF2	elongation factor 2	P13639	6.41	96246	R
1316	−1.23	0.039	SND1	staphylococcal nuclease domain-containing protein 1	Q7KZF4	6.74	102618	CY, N
2647	−1.2	0.04	IMMT	MICOS complex subunit MIC60	Q16891	5.71	79830	M
3165	1.64	0.035	FUBP1	far upstream element-binding protein 1 isoform 2	Q96AE4	7.18	67690	N
6567	1.7	0.05	CLIC4	intracellular chloride channel p64H1	Q9Y696	5.44	28829	CY, Me
7225	5.27	0.049	UCHL1	ubiquitin carboxyl-terminal hydrolase isozyme L1	P09936	5.33	25167	CY
**MMLC** ***versus*** **control (n = 8)**								
**Spot**	**Fold** ^**a**^	***p*** **value**	**Gene**	**Protein**	**Accession**	**pI** ^**b**^	**MW** ^**b**^	**Localization**
6786	−3.99	0.041	GCLM	glutamate–cysteine ligase regulatory subunit	P48507	5.69	31050	CY
5687	−2.24	0.05	LASP1	LIM and SH3 domain protein 1 isoform a	Q14847	6.61	30097	CY, CS
5474	−1.64	0.028	ANXA1	annexin A1	P04083	6.57	38918	CY, N
6322	−1.54	0.048	MAP1B	microtubule-associated protein 1B	P46821	4.73	271651	CY, CS
5298	−1.5	0.012	ANXA1	annexin A1	P04083	6.57	38918	CY, N
5341	−1.46	0.045	ANXA1	annexin A1	P04083	6.57	38918	CY, N
5361	−1.32	0.043	ANXA1	annexin A1	P04083	6.57	38918	CY, N
5390	−1.2	0.023	ANXA1	annexin A1	P04083	6.57	38918	CY, N
5272	−1.2	0.021	ANXA1	annexin A1	P04083	6.57	38918	CY, N
6873	−1.2	0.019	RPL7	ribosomal protein L7	P18124	10.6	29221	R
3472	1.2	0.0005	LMNA	lamin A/C, partial	P62241	7.7	79805	N
4684	1.27	0.0095	PAI1	plasminogen activator inhibitor 1, partial	P05121	6.68	45102	Secr.
5438	2.16	0.021	ANXA2	annexin A2 isoform 2	P07355	7.57	38808	BMe

Differentially represented proteins in: 1) hCFs exposed to cardiotoxic LC (CardioLC) *versus* untreated cells (control); 2) hCFs exposed to CardioLC *versus* non cardiotoxic LC-exposed cells (MMLC) and 3) hCFs exposed to MMLC *versus* control. ^a^Fold is the ratio between the protein expression level of hCFs exposed to CardioLC and the expression level of control or hCFs exposed to MMLC. ^b^Theoretical pI and MW. CY, cytoplasm; N, nucleus; M, mitochondrion; CS, cytoskeleton; R, ribosome; ER, endoplasmic reticulum; Me, cell membrane; Secr., Secreted; BMe, basement membrane.

Using shotgun proteomics, excluding common contaminants, a total of 1,950 distinct proteins were identified. Of these, 1,277 proteins were identified in at least three replicates of Control and 1,278 in at least three replicates of the CardioLC- and of the MMLC-treated hCFs; only these two sets of species were considered for the differential analyses. The highest number of differential proteins was detected in the CardioLC *versus* Control comparison, in which 75 proteins differed significantly according to the stringent criteria described in Methods (*p* value < 0.01, identified with >2 distinct peptides and appearing in > 75% of all replicates) (Table 3). Regarding the CardioLC *versus* the MMLC comparison, 41 proteins were found to be differentially represented (Table 3). Notably, 34 of these were common to the CardioLC *versus* Control comparison, all but one (Vimentin) with the same direction of change (Table 3). In contrast, only 4 differential proteins were identified in the MMLC *versus* Control comparison (Table 3). These data indicate that the molecular profile of hCFs incubated with the control LC did not differ significantly from the non-exposed cells, whereas exposure to the CardioLC translated into significant proteome remodeling (Fig. 2b). Most of the differential proteins in the CardioLCs-treated hCFs were under-represented compared to the Control and/or to the MMLC-treated cells (Fig. 2b). Exceptions are Myosin-9 (MYH9), Heterogeneous nuclear ribonucleoprotein U (HNRNPU), Delta-1-pyrroline-5-carboxylate synthase (ALDH18A1), Cysteine and glycine-rich protein 2 (CSRP2), Eukaryotic translation initiation factor 6 (EIF6), Chloride intracellular channel 4 (CLIC4) and mitochondrial Citrate synthase (CS), which were over-represented. The differential proteins in the CardioLC-treated hCFs are located in various cellular compartments (Table 3). Most of the species are cytosolic (21 proteins) or cytoskeletal (24 proteins) components. However, also the ribosome and the endoplasmic reticulum (5 and 8 differential proteins, respectively), cell membrane (8 proteins), nucleus (7 proteins) and mitochondria (6 proteins) were affected by proteome changes. Moreover, several proteins destined to secretion (Myeloid-derived growth factor, Collagen alpha-2(I) chain, Fibulin-2, Fibronectin) were quantitatively decreased in CardioLC-treated cells. Notably, some proteins were identified exclusively in the CardioLC-treated cells (Supplementary Table S1). These proteins, which were not considered in the differential analysis, include specific entries referred to human immunoglobulin λ light chains, which are known to be internalized by cells^17^.

**Table 3:** Label-free differential analysis.

CardioLC *versus* control (n = 41)(excluding proteins shared with the CardioLC *versus* MMLC comparison)										
Accession	Protein			Gene		Log2FC^a^		*p* value	Localization	Notes
Q969H8	Myeloid-derived growth factor			MYDGF		−0.717		1.08E-04	ER, Secr.	
O00244	Copper transport protein ATOX1			ATOX1		−0.613		5.49E-4	CY	
Q9BVC6	Transmembrane protein 109			TMEM109		−0.586		4.21E-6	N	
O60493	Sorting nexin-3			SNX3		−0.564		9.25E-03	CY	
P52907	F-actin-capping protein subunit alpha-1			CAPZA1		−0.526		6.88E-04	CS	
Q9NVA2	Septin-11			SEPT11		−0.493		6.20E-04	CS	
P39019	40 S ribosomal protein S19			RPS19		−0.476		7.78E-03	N, R	
P68104	Elongation factor 1-alpha 1			EEF1A1		−0.443		2.47E-03	CY, N	
P62269	40 S ribosomal protein S18			RPS18		−0.442		5.22E-04	R	*
Q6UVK1	Chondroitin sulfate proteoglycan 4			CSPG4		−0.430		4.15E-03	Me	
Q15436	Protein transport protein Sec. 23 A			SEC. 23 A		−0.415		9.55E-04	ER	
P46782	40 S ribosomal protein S5			RPS5		−0.414		7.23E-04	R	
Q9NZN4	EH domain-containing protein 2			EHD2		−0.402		2.68E-03	Me	
Q9NZM1	Myoferlin			MYOF		−0.371		3.04E-07	Me	
P13674	Prolyl 4-hydroxylase subunit alpha-1			P4HA1		−0.355		1.77E-03	ER	
P24539	ATP synthase subunit b, mitochondrial			ATP5F1		−0.348		8.27E-03	M	
Q14192	Four and a half LIM domains protein 2			FHL2		−0.345		1.05E-03	N	
P50454	Serpin H1			SERPINH1		−0.340		9.17E-04	ER	
O75083	WD repeat-containing protein 1			WDR1		−0.339		7.56E-04	CS	
P11940	Polyadenylate-binding protein 1			PABPC1		−0.327		9.25E-03	CY	
P18206	Vinculin			VCL		−0.310		1.08E-06	CS	
O43852	Calumenin			CALU		−0.306		3.14E-03	ER	
P05388	60 S acidic ribosomal protein P0			RPLP0		−0.304		7.55E-03	R	
Q15019	Septin-2			SEPT2		−0.298		1.86E-04	CS	
P06733	Alpha-enolase			ENO1		−0.269		4.93E-03	CY	
P04792	Heat shock protein beta-1			HSPB1		−0.257		8.27E-03	CY	
P25705	ATP synthase subunit alpha, mitochondrial			ATP5A1		−0.255		1.79E-03	M	
Q9P2E9	Ribosome-binding protein 1			RRBP1		−0.255		4.96E-04	ER	
Q96AC1	Fermitin family homolog 2			FERMT2		−0.254		5.87E-03	CS, Me	
P46821	Microtubule-associated protein 1B			MAP1B		−0.254		1.04E-03	CS	
P36578	60 S ribosomal protein L4			RPL4		−0.253		6.57E-04	R	
Q16658	Fascin			FSCN1		−0.250		8.14E-03	CS	
P78371	T-complex protein 1 subunit beta			CCT2		−0.227		2.43E-03	CY	
P14625	Endoplasmin (Heat shock protein 90 kDa)			HSP90B1		−0.221		3.24E-04	ER	
P55084	Trifunctional enzyme subunit beta, mitochondrial			HADHB		−0.220		5.23E-03	M	
P26038	Moesin			MSN		−0.216		1.05E-03	Me	
P30101	Protein disulfide-isomerase A3			PDIA3		−0.213		7.06E-03	ER	
Q14204	Cytoplasmic dynein 1 heavy chain 1			DYNC1H1		−0.139		4.85E-03	CS	
P35579	Myosin-9			MYH9		0.182		5.71E-04	CS	
Q00839	Heterogeneous nuclear ribonucleoprotein U			HNRNPU		0.398		4.45E-06	N	
P54886	Delta-1-pyrroline-5-carboxylate synthase			ALDH18A1		0.994		4.06E-03	M	
**CardioLC** ***versus*** **control and** ***versus*** **MMLC (n** **=** **34)**										
			**CardioLC** ***vs*** **MMLC**		**CardioLC** ***vs*** **control**					
**Accession**	**Protein**	**Gene**	**Log2FC** ^**a**^	***p*** **value**	**Log2FC** ^**a**^		***p*** **value**		**Local**	**Notes**
Q86VP6	Cullin-associated NEDD8-dissociated protein 1	CAND1	−1.041	5.58E-04	−1.009		1.74E-03		CY	=
P08670	Vimentin	VIM	−1.029	0.00E + 00	0.531		3.50E-07		CS	≠ ***
P61586	Transforming protein RhoA	RHOA	−0.815	7.94E-03	−0.802		4.93E-03		Me	=
Q9UK76	Hematological and neurological expressed 1 protein	HN1	−0.726	7.94E-03	−0.870		8.54E-05		N	=
P21589	5’-nucleotidase	NT5E	−0.466	2.51E-04	−0.335		5.30E-03		Me	=
P21964	Catechol O-methyltransferase	COMT	−0.464	2.11E-03	−0.414		3.91E-03		CY	=
Q14847	LIM and SH3 domain protein 1	LASP1	−0.415	4.33E-04	−0.540		1.08E-06		CS	=
P60891	Ribose-phosphate pyrophosphokinase 1	PRPS1	−0.340	3.62E-04	−0.399		6.64E-06		CY	=
P08123	Collagen alpha-2(I) chain	COL1A2	−0.339	1.46E-03	−0.334		1.74E-03		Secr.	=
Q00341	Vigilin	HDLBP	−0.338	2.37E-04	−0.354		2.23E-05		CY	=
Q14764	Major vault protein	MVP	−0.326	1.06E-03	−0.279		2.64E-03		CY	=
Q96HC4	PDZ and LIM domain protein 5	PDLIM5	−0.310	1.51E-03	−0.321		7.23E-04		CY	=
P98095	Fibulin-2	FBLN2	−0.303	9.72E-04	−0.261		5.81E-03		Secr.	=
P08758	Annexin A5	ANXA5	−0.283	8.73E-03	−0.276		5.81E-03		CY, Me	=
O60701	UDP-glucose 6-dehydrogenase	UGDH	−0.272	2.17E-03	−0.261		2.89E-03		CY	=
P02751	Fibronectin	FN1	−0.271	5.58E-04	−0.325		1.25E-04		Secr.	=
Q00610	Clathrin heavy chain 1	CLTC	−0.269	1.43E-05	−0.261		3.23E-06		Me	=
P27816	Microtubule-associated protein 4	MAP4	−0.265	9.36E-04	−0.223		3.87E-03		CS	=
P08133	Annexin A6	ANXA6	−0.263	8.15E-03	−0.316		4.07E-04		CY	=
Q13642	Four and a half LIM domains protein 1	FHL1	−0.261	4.83E-03	−0.391		7.55E-07		CY	=
P15924	Desmoplakin	DSP	−0.258	7.56E-05	−0.316		7.72E-08		CS	=
P13639	Elongation factor 2	EEF2	−0.246	3.00E-03	−0.292		7.19E-05		CY, N	=
P07355	Annexin A2	ANXA2	−0.239	2.87E-03	−0.252		6.31E-04		Me	=
Q05682	Caldesmon	CALD1	−0.230	1.43E-05	−0.235		2.75E-06		CS	=
P21333	Filamin-A	FLNA	−0.226	3.16E-08	−0.265		8.11E-12		CS	=
Q14315	Filamin-C	FLNC	−0.216	2.19E-04	−0.246		3.20E-06		CS	=
O43707	Alpha-actinin-4	ACTN4	−0.201	2.37E-04	−0.304		2.63E-10		CS	=
Q15149	Plectin	PLEC	−0.199	3.54E-05	−0.295		1.71E-12		CS	=
P46940	Ras GTPase-activating-like protein IQGAP1	IQGAP1	−0.194	2.15E-03	−0.256		3.42E-06		CS, Me	=
P22314	Ubiquitin-like modifier-activating enzyme 1	UBA1	−0.191	1.13E-03	−0.284		3.13E-08		CY, N	=
O75369	Filamin-B	FLNB	−0.184	1.43E-05	−0.183		3.20E-06		CS	=
Q9Y490	Talin-1	TLN1	−0.156	1.60E-04	−0.245		1.73E-11		CS	=
Q09666	Neuroblast differentiation-associated protein AHNAK	AHNAK	−0.143	1.07E-04	−0.196		2.74E-09		N	=
Q16527	Cysteine and glycine-rich protein 2	CSRP2	0.930	1.60E-04	1.031		5.73E-05		N	=
**CardioLC** ***versus*** **MMLC (n = 7) (excluding proteins shared with the CardioLC** ***versus*** **Control comparison)**										
**Accession**	**Protein**			**Gene**	**Log2FC** ^**a**^		***p*** **value**		**Localization**	**Notes**
P27144	GTP:AMP phosphotransferase AK4, mitochondrial			AK4	−1.156		4.33E-04		M	
P51911	Calponin-1			CNN1	−0.365		6.45E-03		CS	
P02545	Prelamin-A/C			LMNA	−0.348		5.31E-05		N	
P13797	Plastin-3			PLS3	−0.318		7.94E-03		CY	
Q13813	Spectrin alpha chain, non-erythrocytic 1			SPTAN1	−0.298		3.62E-04		CS	**
P56537	Eukaryotic translation initiation factor 6			EIF6	0.501		1.13E-03		CY	
O75390	Citrate synthase, mitochondrial			CS	0.944		9.18E-03		M	
**MMLC** ***versus*** **control (n = 4)**										
**Accession**	**Protein**			**Gene**	**Log2FC** ^**a**^		***p*** **value**		**Localization**	**Notes**
P62269	40 S ribosomal protein S18			RPS18	−0.476		4.30E-04		R	*
Q01082	Spectrin beta chain, non-erythrocytic 1			SPTBN1	0.240		3.94E-03		CS	
Q13813	Spectrin alpha chain, non-erythrocytic 1			SPTAN1	0.266		4.34E-03		CS	**
P08670	Vimentin			VIM	1.561		0.00E + 00		CS	***

Differentially represented proteins in: 1) hCFs exposed to cardiotoxic LC (CardioLC) *versus* untreated cells (control); 2) hCFs exposed to CardioLC *versus* non cardiotoxic LC-exposed cells (MMLC); 3) common to these two comparisons and 4) hCFs exposed to MMLC *versus* control. ^**a**^Log2 Fold Change, whose negative values indicate under-represented proteins and positive values indicate over-represented proteins. =Same direction of change in the CardioLC *versus* MMLC and in the CardioLC *versus* control comparisons; ≠opposite direction of change; *present in the CardioLC *versus* control and in the MMLC *versus* control comparisons; **present in the MMLC *versus* control and CardioLC *versus* MMLC comparisons; ***present in all comparisons. CY, cytoplasm; N, nucleus; M, mitochondrion; CS, cytoskeleton; R, ribosome; ER, endoplasmic reticulum; Me, cell membrane; Secr., Secreted.

**Figure 3:**
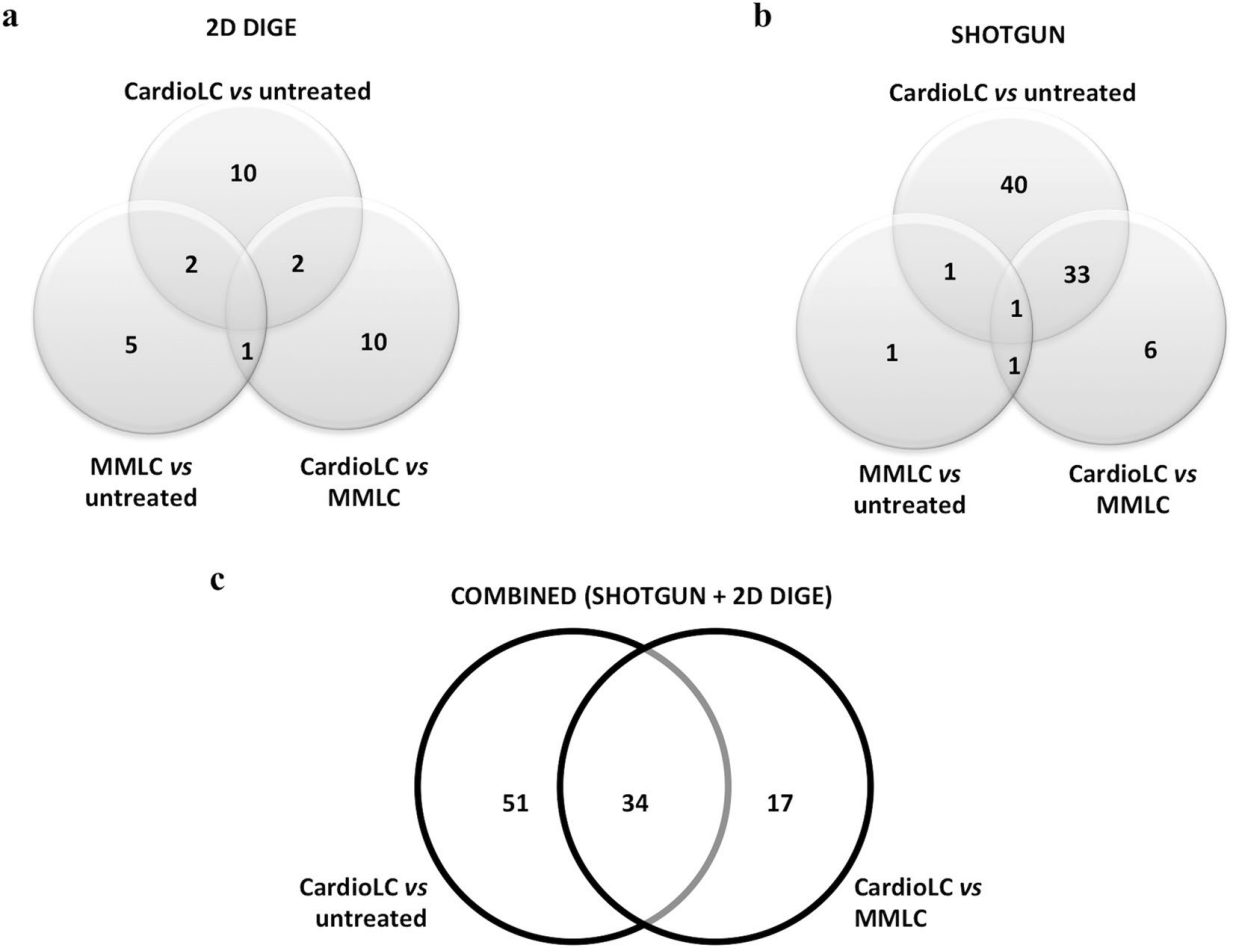
Venn diagrams summarizing the identified differentially represented proteins. The proteins specific for each comparison (CardioLC *versus* MMLC, CardioLC *versus* untreated, MMLC *versus* untreated) and those shared by the different comparisons are shown for both (**a**) 2D DIGE and (**b**) shotgun analyses. (**c**) Venn diagram summarizing the identified differential proteins in CardioLC-treated cells, compared to MMLC-treated cells and to untreated cells, by combining the results of the shotgun and 2D DIGE analyses.

Combining the results of the two proteomic methodologies, 85 differentially expressed proteins were found in the CardioLC *versus* Control comparison and 51 in the CardioLC *versus* the MMLC comparison (Fig. 3). Of all these proteins, 4 (LIM and SH3 domain protein 1 isoform a, Talin, heat shock protein 27 and microtubule-associated protein 1B) were identified in the CardioLC *versus* control and 3 (Lamin isoform A, Caldesmon isoform 2, Elongation factor 2) in the CardioLC *versus* the MMLC comparisons irrespective of the proteomic technique (Table 4). In all cases, the direction of change of these proteins in the two studies was concordant. This observation clearly shows that the two approaches provide highly complementary results and underlines the importance of exploring the proteome with multiple approaches.

**Table 4:** Differentially represented proteins identified by both the 2D DIGE and the shotgun differential analyses.

**CardioLC** ***versus*** **control cells**		
Gene name	Protein name	UniProt
LASP1	LIM and SH3 domain protein 1 isoform a	Q14847
TLN	Talin	Q9Y490
HSPB1	Heat shock protein 27	P04792
MAP1B	Microtubule-associated protein 1B	P46821
**CardioLC** ***versus*** **MMLC**		
LMNA	Lamin isoform A	P02545
CALD1	Caldesmon isoform 2	Q05682
EEF2	Elongation factor 2	P13639

### Bioinformatics analysis of differentially represented proteins

We used bioinformatics tools to identify the functional categories and relevant networks and pathways among the differential proteins identified with the two approaches (Fig. 3c). The enriched Gene Ontology (GO) terms according to the DAVID functional annotation tool are listed in Table 5. Terms related to cytoskeleton, contractile fibers and ribosomes were the most relevant terms involving the differential proteins in the CardioLC-treated hCFs; in particular, the cytoskeleton was involved in both the comparison with the MMLC-treated hCFs and with control cells (Table 5). Ingenuity Pathways Analysis showed the relations existing among the differential proteins of the CardioLC-treated hCFs. In particular, considering the comparison between the CardioLC-treated hCFs and untreated cells, two high-score multidirectional interaction networks were identified that were associated, respectively, with “Cancer, Cell Death and Survival, Organismal Injury and Abnormalities” (score = 78) and “Cell Morphology, Cellular Assembly and Organization, Cellular Function and Maintenance” (score = 67) (Supplementary Figure S1a and b). Regarding the CardioLC *versus* MMLC comparison, differential proteins were connected in a high-score network, associated with “Developmental Disorder, Hereditary Disorder, Organismal Injury and Abnormalities” (score = 74) (Supplementary Figure S1c). Red and green nodes in Supplementary Figure S1 indicate differential proteins specific for the CardioLC *versus* control and the CardioLC *versus* the MMLC comparisons, respectively, whereas blue nodes indicate differential proteins common to both comparisons.

**Table 5:** Functional annotation analysis of differentially represented proteins in hCFs exposed to CardioLC *versus* control cells and MMLC-exposed cells.

GO	Proteins	*p* value
**CardioLC** ***versus*** **control cells**		
Actin cytoskeleton organization	TLN1, ACTN4, CALD1, FERMT2, FSCN1, CAPZA1, RHOA, MYH9, EHD2, FLNB, FLNA	3.9 × 10^−7^
Contractile fiber	PDLIM5, FHL2, HSPB1, FLNC, FLNB, VCL, ENO1, PLEC	5.6 × 10^−6^
Cytosolic ribosome	RPS18, RPS19, RPLP0, RPL4, RPS5, RPS8	9.7 × 10^−5^
Adherens junction	TLN1, LASP1, FERMT2, FHL2, DSP, MYH9, VCL	2.5 × 10^−4^
Collagen fibril organization	P4HA1, COL1A2, SERPINH1, ANXA2	5.2 × 10^−4^
Cytoplasmic vesicle	ANXA6, SEC. 23 A, HSP90B1, GANAB, PDIA3, ACTN4, CLTC, MYOF, FN1, ANXA2, CALU	3.4 × 10^−3^
Regulation of cytoskeleton organization	MAP1B, CAPZA1, RHOA, MAP4, MAPRE1	6.5 × 10^−3^
Guanyl ribonucleotide binding	EEF1A1, SEPT2, RHOA, EEF2, EHD2, SEPT11, PRPS1	1.9 × 10^−2^
**CardioLC** ***versus*** **MMLC**		
Cytoskeleton	EIF6, TLN1, ACTN4, PDLIM5, CALD1, VIM, LMNA, DPYSL2, FLNC, FLNB, FLNA, IQGAP1, LASP1, CLIC4, RHOA, DSP, MAP4, SPTAN1, PLEC	1.0 × 10^−7^
Cytoplasmic vesicle	ANXA6, ACTN4, CLIC4, SND1, CLTC, FN1, ANXA2	1.8 × 10^−2^

### Verification of selected candidates via western blotting

**Figure 4:**
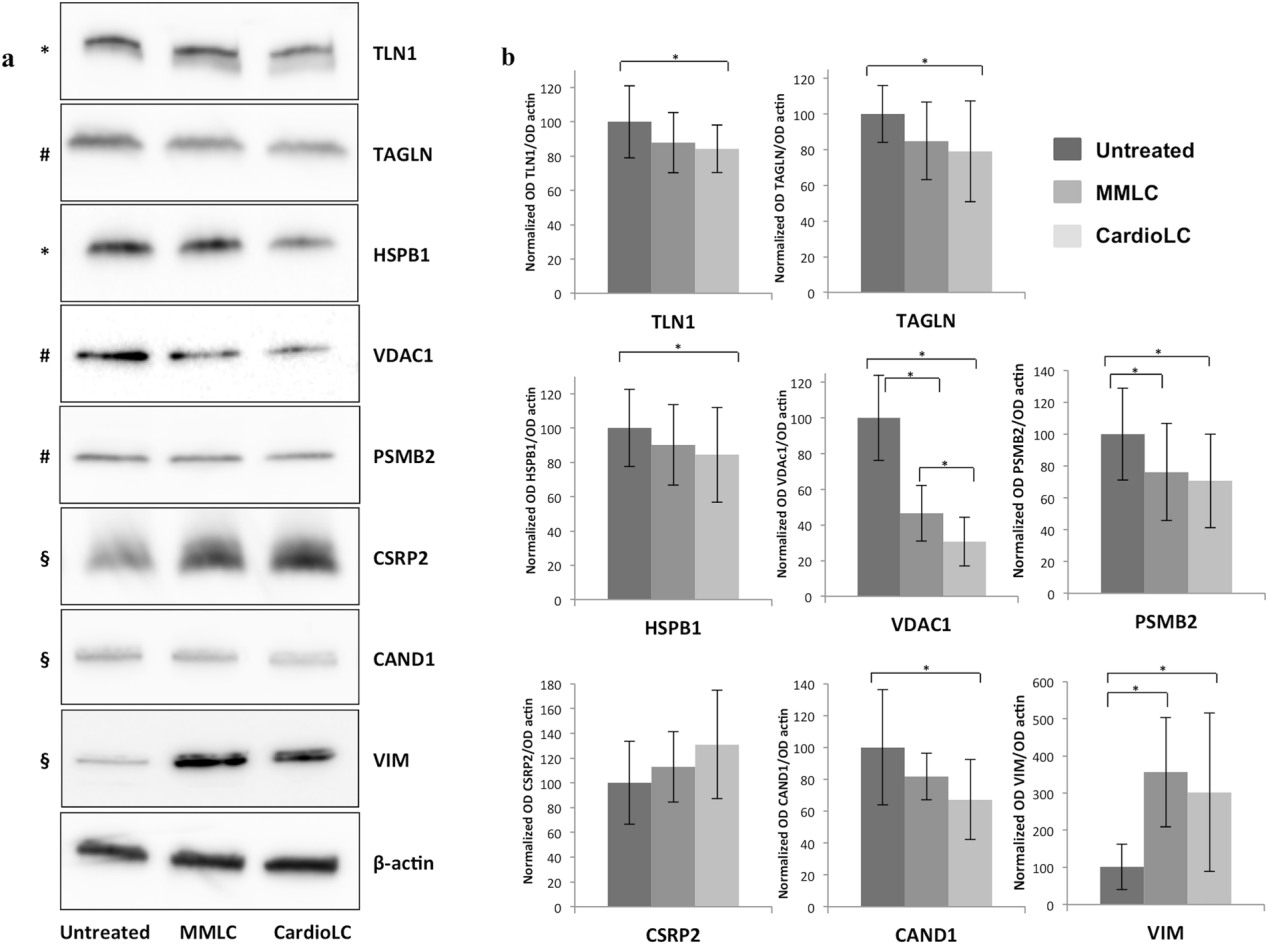
Verification of differentially represented proteins using western blotting. (**a**) Western blot images of a subset of selected proteins identified using 2D DIGE (#), label-free analysis (§) or both approaches (*). Cropped images of a representative replicate per protein are shown; full-length blots are shown in Supplementary Figure S2. Proteins displayed in this image were separately analyzed. 10 μg of protein extracts from hCFs in each experimental condition (exposed for 24 h with CardioLC, MMLC or untreated) were separated by SDS-PAGE and analyzed by immunoblot. (**b**) Densitometric and statistical analyses of western blot signals were performed using three biological replicates, each one considered as the average of at least two technical replicates. The graphs display the ratio (average values; bars represent standard deviations) between the signal of each protein and the corresponding β-actin, normalized against the average of the corresponding control cells. *p value < 0.05.

Among the differentially represented proteins identified by 2D DIGE and label-free analysis, or both, 8 species were selected for independent validation by western blotting (Fig. 4). These proteins are: Talin (TLN) and Heat shock protein 27 (HSPB1) (found to be reduced in the CardioLC-treated hCFs by both proteomic approaches), Transgelin (TAGLN), Porin 31HM (VDAC1) and Proteasome subunit beta type-2 (PSMB2) (found to be reduced in the CardioLC-treated hCFs by 2D DIGE), Cysteine and glycine-rich protein 2 (CSRP2) and Cullin-associated NEDD8-dissociated protein 1 (CAND1) (respectively, increased and decreased in the CardioLC-treated cells according to LC-MS/MS), and Vimentin (VIM) (increased in the CardioLC- and MMLC-treated cells according to LC-MS/MS). The densitometry signal of each candidate (representative images are shown in Fig. 4a) was normalized in all cases on β-actin, and expressed as relative to the mean of controls (Fig. 4b). Notably, β-actin was not identified as being differentially represented by either proteomic approach. As shown in Fig. 4, the western blot analysis confirmed in all cases the trend of variation indicated by proteomics. The statistically significant differences in the protein levels are reported for those cases in which the *p* values, calculated from the densitometry results, were < 0.05.

### Evaluation of changes in cellular physiology

**Figure 5:**
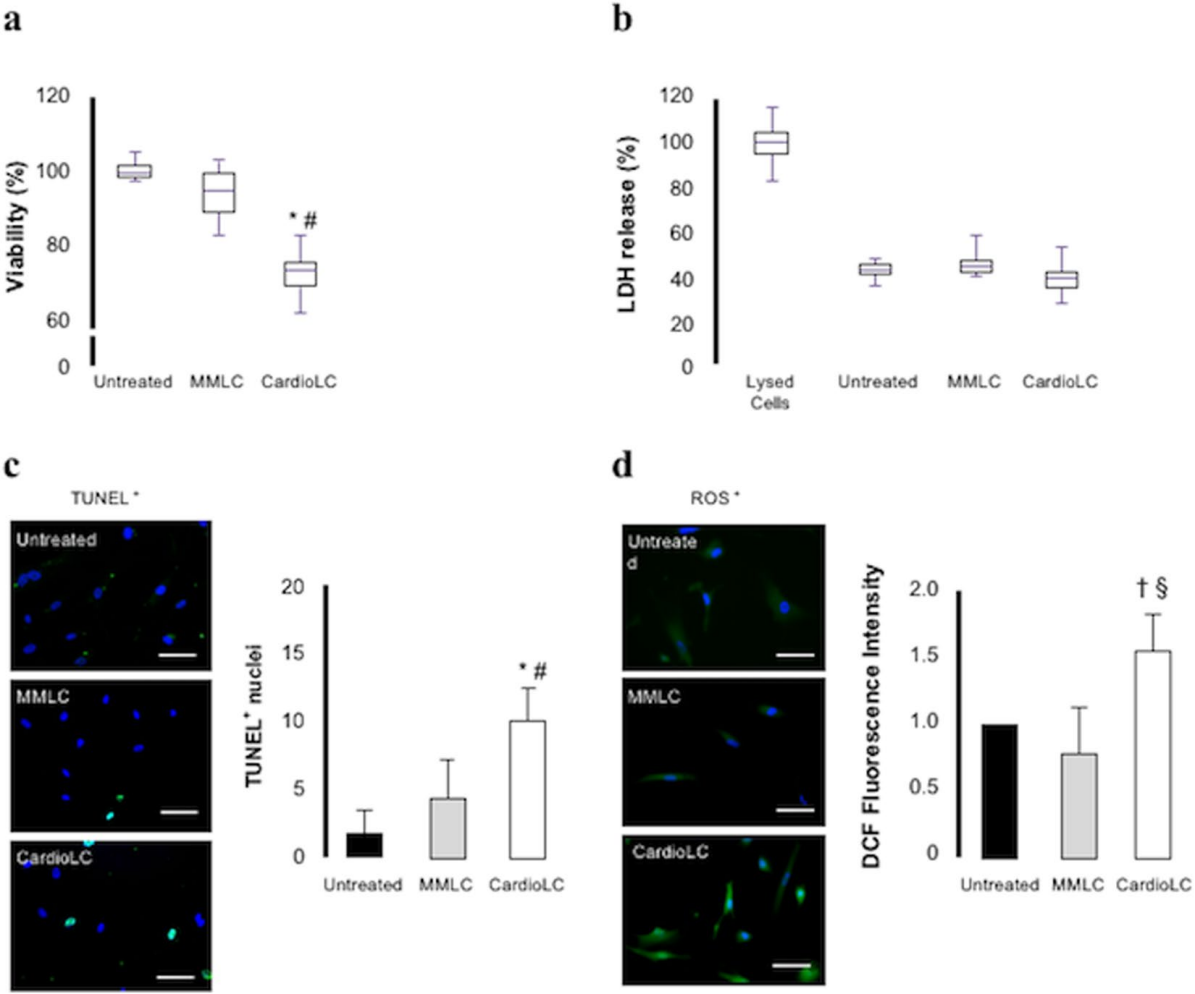
Evaluation of change in cellular physiology. (**a**) Cell viability was significantly reduced in hCFs exposed for 24 h to CardioLC, compared to MMLC-treated and untreated ones. (**b**) Levels of LDH did not differ between hCFs exposed to MMLC or to CardioLC from with untreated hCFs. (**c**) Quantitative analysis and representative images of TUNEL assay of hCFs untreated and exposed to MMLC and CardioLC. Nuclei in blue; TUNEL^+^ cells in green. (**d**) Dichlorofluorescein (DCF) intensity and representative microscopy images of hCFs loaded with ROS-sensitive fluorophore dichlorofluorescein-diacetate (green). All assays were performed using five biological replicates, each one in three technical replicates. Scale bar: 100 µm. *p value < 0.001 *versus* untreated; ^#^ *p* value < 0.001 *versus* MMLC; ^†^ *p* value < 0.05 *versus* untreated; ^§^ *p* value < 0.01 *versus* MMLC.

Exposure to the CardioLC used for the proteomic analyses significantly reduced hCF viability compared with the MMLC-treated and with the untreated cells (*p* values < 0.001 for both comparisons) (Fig. 5a). This result was further confirmed by testing the effects on viability of 3 additional cardiotropic LCs from patients with cardiac AL amyloidosis, and two additional non-amyloidogenic LCs from multiple myeloma patients (Supplementary Figure S3). Exposure to the various MMLCs did not significantly alter cell viability (*p* value = n.s. *versus* untreated cells) (Fig. 5a). Levels of LDH in the culture medium did not differ significantly between treated and untreated cells. This suggests that the cell membrane is not damaged after exposure for 24 hours to the CardioLC (Fig. 5b, referred to CardioLC-1 and MMLC-2). On the contrary, TUNEL assay documented that the exposure to CardioLC significantly increased nuclear fragmentation by 3.1 fold (*p* value < 0.001) compared with untreated cells, while exposure to the MMLC did not significantly increase cell apoptosis (*p* value = ns *versus* untreated cells) (Fig. 5c, referred to CardioLC-1 and MMLC-2).

To explore the mechanism leading to cell apoptosis, we measured ROS production, and found that it was significantly increased after exposure to the CardioLC, compared with both the MMLC (+100%, *p* value < 0.01) and untreated hCFs (+56%, *p* value < 0.05) (Fig. 5d, referred to CardioLC-1 and MMLC-2). The results obtained using the commercial fibroblasts were comparable with those obtained with hCFs isolated from patients.

### Western blot assessment of protein levels in hCFs incubated with other LCs

**Figure 6:**
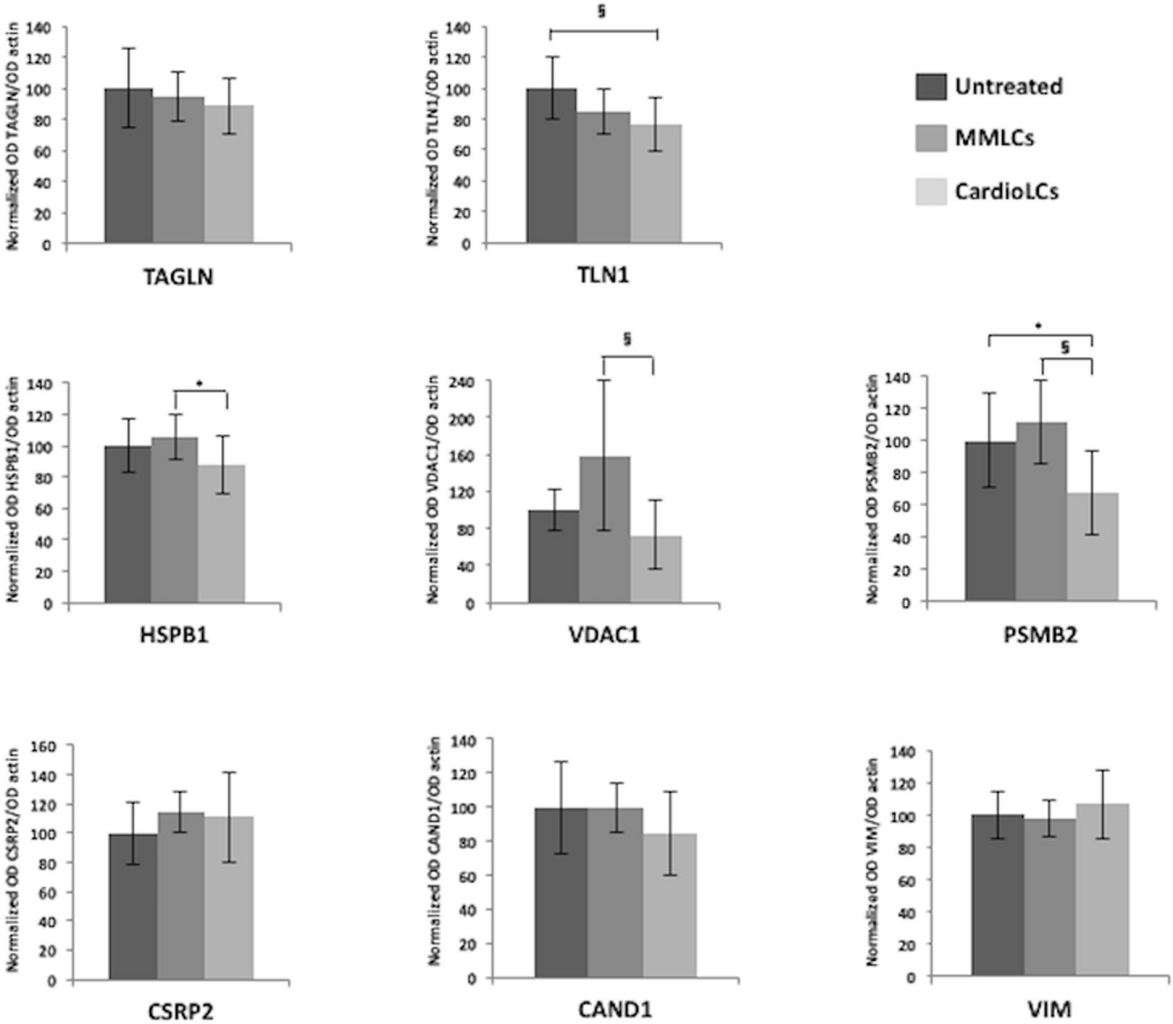
Verification of differentially represented proteins in hCFs incubated with other LCs using western blotting. Densitometric and statistical analysis of western blot signals were performed using two biological replicates (each one in two technical replicates) from hCFs exposed for 24 h to 3 CardioLCs (CardioLC-2, CardioLC-3 and CardioLC-4 in Table 1) and compared to 2 MMLCs (MMLC-1 and MMLC-3 in Table 1) or to untreated cells. The graphs show the ratio (average values; bars represent standard deviations) between the signal of each protein and the corresponding β-actin, normalized against the average of the corresponding control cells. *p value < 0.05; ^§^ *p* value < 0.0167.

The eight cellular proteins listed in the validation section (TLN, HSPB1, TAGLN, VDAC1, PSMB2, CSRP2, CAND1 and VIM) were also assessed by western blotting in hCFs incubated with each of the LCs listed in Table 1, and in untreated cells. The results are reported in Figs 6 and S4. When the new CardioLCs and MMLCs were analyzed collectively, the changes coincided with those originally induced by CardioLC-1, and were statistically significant in 4 out of the 8 analyzed proteins (TAL, VADC1, PSMB2 and HSPB1). The trend was confirmed in TAGLN, CSRP2 and CAND1, although the differences *versus* controls were not statistically significant. In the case of VIM, a slight, albeit not significant, increase was induced by the new CardioLCs, and thus did not reproduce the original pronounced rise. As shown in Figure S4, the various LCs were highly heterogeneous: 6 proteins were significantly affected by treatment with CardioLC-2 (TAL, TAGLN, CAND1, PSMB2, VDAC1 and HSPB1), 3 by CardioLC-3 (TAL, PSMB2 and VDAC1) and 2 by CardioLC-4 (CSRP2 and PSMB2) (Figure S4). Notably, in this set of experiments, the results obtained with CardioLC-1 and MMLC-2 (included as internal controls) matched those observed in the proteomic study, apart from a few minor discrepancies regarding the non-pathogenic LC (an increase rather than a decrease in VDAC1, and a lack of increase in VIM). These discrepancies, not observed for the pathogenic LC, may indicate differences in the response of distinct batches of hCFs to some stimuli.

## Discussion

In this study we sought to describe the molecular changes occurring in hCFs exposed *in vitro* to exogenous LCs causing AL cardiomyopathy, through the characterization of their cellular proteome. As mentioned, the fact that the pre-fibrillar precursors possess intrinsic toxicity is suggested by solid clinical evidence and is corroborated by studies on cultured cells and animal models of LC proteotoxicity^9–19^, including those presented herein. Among these systems, cell cultures are the only examples in which the human homologs can be studied and are therefore important tools with which to investigate LC pathogenicity in a physiologically relevant environment. Cardiac fibroblasts are the predominant cell type in the human heart: they play a crucial role in matrix organization and in tissue physiology, and are plausible important contributors to the overall dysfunction in AL cardiomyopathy^20,21^. These cells internalize cardiotropic LCs *in vitro*, thereby resulting in changes in proteoglycan production and ultimately in cell damage^15–17^. Moreover, being primary cells, hCFs are more closely representative of the *in vivo* physiology than are cell lines, and are in practice the only human cardiac cell type that can be obtained *ex-vivo* in significant amounts and that can be stably propagated (in contrast to terminally differentiated adult cardiomyocytes). Our cell-based analyses indicate that prefibrillar cardiotropic LCs cause oxidative stress and affect the viability of hCFs by activating apoptotic mechanisms. These results are in line with what has been documented in rodent cardiomyocytes^12,13,22^, in the pharynx of *C*. *elegans* ^14^, and in human cardiac cell lines^19^.

Here we describe the perturbations at molecular level caused by the representative cardiotropic LC investigated, singling-out the component of damage caused by the soluble precursor. We used two independent proteomic methodologies in the attempt to identify high-confidence changes and to expand the proteome coverage. Our proteomic data confirm the effectiveness of combining the two approaches as previously reported in another system^23^. We also used two types of controls: cells grown in parallel to LC-treated cells but not exposed to LCs, and cells incubated with a representative non-amyloidogenic and non-cardiotoxic LC (MMLC)^14,17^. Quantitative alterations in a large number of specific proteins were identified in cells treated with the cardiotoxic LCs, which indicates pronounced proteome remodeling. In accordance with the cellular assays, the changes in cells treated with the control LCs were significantly more limited thereby confirming that, although both LCs are internalized, only the toxic ones cause a significant subversion of the cellular environment^14,17^. The main classes of differential proteins, grouped according to their functional category and analyzed in the context of the documented physiology alterations, are discussed below.

An intriguing observation of our study concerns the role of ROS, whose levels have been reported to be significantly higher in hCFs treated with cardiotropic LCs than in control cells^3,14,22,24,25^. Indeed, the increased levels of three of the proteins we identified suggest a response to oxidative stress in these hCFs. These proteins are: (1) CLIC4, whose levels are increased by ROS in fibroblasts thereby leading to TGF-β1-induced differentiation into myofibroblasts^26^; (2) HNRNPU, which protects cells after oxidative stress by excision repair of oxidized DNA bases^27^; and (3) ALDH18A1, which belongs to the mitochondrial family of aldehyde dehydrogenases, known to act as redox scavengers^28^. A special case is the Copper transport protein ATOX1, which is a copper chaperone that protects against oxidative stress and was reduced in CardioLC-treated hCFs. In the light of the recent evidence that copper is directly involved in H_2_O_2_ generation by amyloidogenic cardiotropic LCs^25^, it is tempting to speculate that the ATOX1 decrease plays a functional role in the genesis and perpetuation of damage. It is also noteworthy that other differential proteins documented herein, which are not directly involved in the antioxidant response, have been previously shown to be quantitatively (mostly reduced, in line with our evidence) and qualitatively altered in cells upon oxidative injury. These include ATP synthase, VDAC, UCHL-1, proteasome subunits, ion channels, protein transporters, chaperones and various ribosomal and structural proteins^29–32^, thereby indicating that proteome remodeling after oxidative stress is a complex phenomenon that translates into alteration of cell compartments and functions. Moreover, although we did not identify components of the core apoptotic signaling pathway, we found, by proteomics, many species that have been reported to be altered in apoptotic cells^33^, namely MICOS complex subunit MIC60 (IMMT), CLIC4, dihydropyrimidinase-related protein 2 isoform 2 (DPYSL2), Ubiquitin-like modifier-activating enzyme 1 (UBA1) and HSPB1. Some of these species are discussed more in detail in the following paragraphs. In agreement with proteomic studies related to apoptosis, our data also revealed protein alteration of potential caspase substrates. It should be noted however that we used primary hCF cultures, and thus we cannot exclude a degree of heterogeneity among cells. Therefore, signals from specific subgroups (such as cells already in apoptosis) are averaged with the rest of the population, and we speculate that the observed proteomic alterations are final endpoints that can result from the co-participation of an array of molecular causes and that translate into cell and viability alterations.

A significantly altered compartment in CardioLC-treated hCFs is the cytoskeleton (Table 4), both in terms of structural and of regulatory proteins. This conclusion is supported by bioinformatics, which revealed that most of the identified differential proteins are involved in cell morphology and cellular assembly and organization (Supplementary Figure S1). Interestingly, several members of this compartment are also concordantly altered in patients’ fat tissue (Supplementary Table S3)^34^. Most differentially abundant cytoskeletal proteins are reduced in CardioLC-treated hCFs compared to controls; among these, TLN1 (validated by western blot in Fig. 4) is especially attractive since it was identified by both proteomic methods and is decreased *in vivo* ^34^. TLN1 is a high molecular weight protein involved in connecting cytoskeletal structures to the plasma membrane and that plays a central role in cell-matrix contacts^35^. This protein is necessary for myofibril stability^36^ and its reduction alters cell adhesion and decreases spreading^35^. Interestingly, as shown by our proteomic and western blot data, and in line with an *in vivo* study^34^, the decrease of TLN1, an actin-binding protein, is not associated with the alteration of β-actin protein levels. This coincides with the observation that a decrease of TLN1 does not affect the level of cytoskeletal proteins such as vinculin and actin^35^, whereas β-actin reduction translates into decreased TLN1^37^.

Our study also shows the alteration of several non-structural species involved in cell organization, differentiation and proliferation, including CSRP2 (Fig. 4), Neuroblast differentiation-associated protein AHNAK and Prelamin A/C (reduced also in tissues, Supplementary Table S3)^34^, Transforming protein RhoA and multiple zinc finger motif-containing proteins (PDLIM5, FHL1 and FHL2), as well as proteins with signaling properties, such as Hematological and neurological expressed 1 protein (HN1), Ras GTPase-activating-like protein IQGAP1 and Ras suppressor protein 1 (RSU1). Speculatively, the reduced abundance of structural and regulatory proteins may be correlated with a defect in reprogramming or differentiation in response to specific stimuli that may influence cell viability and tissue organization within the heart^38–40^. In contrast with the other cytoskeletal proteins, we found that VIM, an intermediate filament protein expressed in mesenchymal cells, was increased in hCFs exposed to both LCs (Fig. 4). However, this trend was not reproduced in hCFs from different batches and treated with distinct LCs (Figs 6 and S4). Given this high variability, it is not possible, at this stage, to hypothesize a role for this protein in this context.

In accordance with the trend observed in affected tissues^34,41^, our dataset showed the reduction of several cellular species involved in protein folding and heat shock response (namely, HSPB1, validated in Fig. 4, Serpin H1 [SERPINH1], Calumenin, T-complex protein 1 subunit beta [CALU], Protein disulfide-isomerase A3 [PDIA3]) and Heat shock protein 90 kDa [HSP90B1]), and of proteins belonging to the ubiquitin-proteasome system, such as PSMB2 and CAND1 (Fig. 4), Proteasome subunit HSPC (PSMA7), Ubiquitin carboxyl-terminal hydrolase isozyme L1 (UCHL1) and UBA1. The observation that cardiac cells do not counteract the presence of misfolding-prone LCs through an increase in protective species is in line with the notion that impaired proteostasis plays a pathogenic role in neurodegenerative protein misfolding diseases^42,43^. It is still unclear, at this point, how an external proteotoxic stimulus can lead to alterations in the quality control compartment; it can be speculated, however, that the quantitative impairment in the protein degradation and folding apparatus reduces the efficiency to cope with these toxic agents, as well as with altered self-proteins thereby creating a vicious circle that perpetuates damage and ultimately compromises the cell.

Our proteomic study included key proteins involved in energy production, such as subunits α and β of ATP synthase (ATP5A1 and ATP5F1, which are under-represented also in AL-affected tissues, Supplementary Table S3)^34^, Trifunctional enzyme subunit beta (HADHB), Voltage-dependent anion-selective channel protein 1 (VDAC1) (Fig. 4), and MICOS complex subunit MIC60 (IMMT). The decrease of these proteins may subvert the mitochondrial function at multiple levels. Mitochondria are indeed emerging as crucial players in protein misfolding diseases^9,10,17,44,45^, and the function of these organelles may be altered through a complex combination of direct^17,46^ and indirect mechanisms^9,10,45^. In a previous study we showed, in particular, that cardiotoxic LCs interact with mitochondrial proteins in hCFs, including VDAC1 and Optic atrophy 1 protein (OPA1)^17^, and that cardiotoxic LC-treated hCFs display mitochondrial ultrastructural changes, especially involving the cristae. The reduction of VDAC1 (an outer mitochondrial membrane protein that allows diffusion of small hydrophilic molecules and possibly plays a role in apoptosis) supports the hypothesis of a dysregulation of mitochondrial transport and of the organelle’s function. Accordingly, we documented the reduction of IMMT, a component of the MICOS protein complex, which plays crucial roles in maintenance of crista junctions and inner membrane architecture^47,48^. These findings support the notion that the complex processes that regulate mitochondrial morphology and cristae structure may be altered in cells exposed to CardioLCs, possibly contributing to the cascade of events that leads to cell dysfunction and trigger apoptotic cascades.

Our bioinformatics analysis showed that the translational apparatus of the cell was also significantly affected by the proteome changes. Overall, 6 distinct proteins belonging to both the large 60 S and the small 40 S subunits were decreased upon treatment with the cardiotoxic LC (Tables 1, 2 and 4). In addition, a set of regulatory proteins important for transcription and translation processes was affected, namely Elongation factors 2 (EEF2) and 1-alpha 1 (EEF1A1), Ribosome-binding protein 1 (RRBP1), Far upstream element-binding protein 1 (FUBP), and Leucyl tRNA synthetase (LARS). These data may indicate an impairment in the protidosynthetic activity of hCFs exposed to the toxic LCs. This “acquired ribosomopathy”^49–51^ in response to misfolding-prone toxic LCs is concordant with the notion that ribosome dysfunction and decreased ribosomal RNA and tRNA levels^52,53^ are early events in the neurodegenerative disorder Alzheimer’s disease suggesting that alterations in protein synthesis contribute to the development of amyloid diseases^52,53^.

In line with our previous *in vivo* study^34^, we found that species involved in the formation and organization of the ECM (Collagen alpha-2(I) chain [COL1A2], Fibulin-2 [FBLN2] and Fibronectin [FN1], and TAGLN) were quantitatively altered. Notably, TAGLN, which was reduced in CardioLC-treated cells (validated in Fig. 4), has been implicated in the suppression of metallomatrix protease-9 (MMP-9)^54^. The latter finding coincides with the notion that MMPs are increased in amyloid cardiomyopathy^55^, and may thus contribute to the disruption of the myocardial ECM homeostasis^56^.

Our data also indicate that molecular trafficking, both vesicle-mediated and membrane transporter-mediated is affected by CardioLC exposure. The involvement of vesicular structures was confirmed by bioinformatics (Table 4), and was related to the reduction of proteins such as Clathrin heavy chain 1 (CLTC) that we observed also in affected tissues (Supplementary Table S3)^34^. The alteration in the import/export and distribution of proteins and other molecules is likely to affect cellular metabolism and the normal processes of cellular interactions with the environment and between different intracellular compartments.

In addition to the proteomic study, we performed a pilot assessment of the generalizability of these results by evaluating the levels of a subset of differential proteins in hCFs treated with distinct cardiotropic and control LCs (Figs 6 and S4). In most of the tested species, changes concordant with those observed in the proteomic study were confirmed upon exposure to other cardiotropic LCs albeit with differences in the level of variation and the number of proteins affected by each amyloidogenic precursor. Overall, the proteins found to be significantly altered in at least half of the cardiotropic LCs tested are TAGLN, CAND1, TLN1, HSPB1, PSMB2 and VDAC1, which supports the existence of shared alterations in mitochondria, cytoskeletal organization and in the protein quality control apparatus. In this context, it should be noted that the proteomic data were obtained in a specific experimental context, and that not all the results may be automatically generalized to all LCs or to distinct experimental conditions. In particular, the proteomic study focused on changes in hCFs exposed to a single cardiotropic LC and a single control lambda LC, and only one subset of proteins was assessed in hCFs exposed to the three new lambda LCs that differ in sequence structure. Given the biochemical diversity of these amyloidogenic proteins, it is very likely that the molecular mechanisms leading to cell toxicity may not be identical across LCs. In addition, we used primary cells, and we cannot exclude that the cells’ responses may vary among human donors, or upon modifications in different culture conditions.

In summary, we characterized molecular alterations occurring in target cells exposed to an extrinsic proteotoxic stimulus^4^, namely, cardiotropic LCs. Taken together, our results indicate that the shared viability impairment caused by the various cardiotropic LCs is associated with changes in the levels of specific proteins in cardiac cells. This finding supports the hypothesis that an altered proteome profile is associated with LC proteotoxicity. The concept that cardiotoxicity due to misfolded pre-fibrillar aggregates may be a more general mechanisms of heart failure, not only limited to amyloidoses, is an emerging topic in cardiovascular research that highlights the importance of characterizing the pathways of intrinsic and extrinsic proteotoxicity^4,57^. Some of our *in vitro* findings have been shown to have parallels *in vivo* ^34^, and quantitative changes in specific proteins may in the future be explored as potential markers of cardiac proteotoxic stress. Moreover, dysregulation of species involved in such crucial functions as cytoskeletal remodeling, mitochondrial activity and metabolism, protein synthesis, quality control and degradation, may play a causative role in the cellular alterations that lead to the clinical picture of AL amyloidosis and targeting these altered pathways may be beneficial in counteracting organ damage in this disease.

## Methods

### Proteins and cells

Monoclonal LCs were isolated as described from urines of patients with cardiac AL amyloidosis and multiple myeloma without amyloidosis (control)^17,58^ (Table 1). Purified proteins were characterized by SDS-PAGE, immunoblotting and mass spectrometry (MS) and assessed for the presence of endotoxins, as previously reported in detail^17,58^. Presence of amyloid deposits and amyloid organ involvement were clinically assessed as recommended^17,58,59^. Primary cardiac fibroblasts (hCFs) from normal human adult heart were both purchased from European Collection of Cell Cultures (Public Health England, #306-05 A) and isolated from human right atrial appendages removed during routine cardiac surgery. Acquisition and use of all human samples for research purposes were approved by the Institutional Review Board of Fondazione IRCCS Policlinico San Matteo Pavia; all methods were performed in accordance with the relevant guidelines and regulations. Written informed consent was received from all subjects. For hCF isolation, heart samples were cut into fragments, washed and partially enzymatically digested with trypsin 0.25% in Phosphate Buffer Saline (PBS). Tissue fragments were placed in fibronectin coated dishes (BD PharMingen™, BD Biosciences, San Jose, CA, USA). After 1 week, stromal-like cells migrated from adherent explants. These cells were harvested few days later and seeded into standard culture dishes. Cells were cultured in serum-additioned medium^17^, until near confluence.

For cell viability assays, cells seeded in 96-wells plates were exposed for 24 h to 5 μM (100 μg/ml) to each of the four CardioLCs or to each of the three MMLCs reported in Table 1. For the proteomic studies, hCFs cultured in75 cm^2^ flasks were incubated with either an amyloidogenic cardiotoxic (CardioLC #1 in Table 1) or a non-amyloidogenic/non-cardiotoxic (MMLC #2 in Table 1) LC (24 h, 5 μM LC). These LCs were selected as representative among those previously tested in our *C*. *elegans* model and in the hCF cultures^14,17^. To validate the proteomic data, we cultured hCFs in 25 cm^2^ flasks, and incubated them with each of the LCs listed in Table 1. Cells not exposed to LCs served as negative control in all assays. Light chains were diluted in the culture medium from a stock dissolved in phosphate buffered saline (PBS); in the unexposed cells, an equivalent volume of PBS was added to the medium. For the proteomics studies, four independent biological replicates were obtained overall, using commercial fibroblasts. Each replicate consisted of the three experimental conditions acquired simultaneously (CardioLC, MMLC, untreated) on the same batch of cells. At the end of the incubation time, cells were washed three times with warm PBS, recovered by scraping and homogenized in modified RIPA buffer (1% NP-40, 0.1% Sodium deoxycholate, 150 mM NaCl, 1 mM EDTA, 50 mM Tris pH 7.5, 1X protease inhibitors)^60^. Cell debris was removed by centrifugation and proteins were quantified using the micro BCA assay (Pierce, Thermo Scientific, Rockford, IL, USA).

### 2D DIGE analysis

#### Sample preparation and electrophoretic separation

The protein samples were precipitated using a 2D Clean-up kit (GE Healthcare) and re-solubilized in lysis buffer containing 7 M Urea, 2 M Thiourea, 30 mM Tris-HCl, pH 7.5, 4% CHAPS and protease inhibitor mix (GE Healthcare, Piscataway, NJ, USA). Protein concentrations were determined using 2D Quant kit (GE Healthcare) following the manufacturer’s instructions. The 2D DIGE experiment was performed using four biological replicates (Supplementary Table S2) and according to the manufacturer’s protocol. Protein extracts (50 μg) from hCFs exposed to CardioLC and MMLC, and from control cells were labeled separately with 400 pmol of Cy3 and Cy5. As internal standard, a mix of equal amounts of all twelve samples under analysis was labeled with Cy2. Labeling reactions were performed as previously described^61^. To avoid differences due to dye-specific protein labeling, we swapped the dyes between the pairs of biological replicates (Supplementary Table S2). The samples were mixed as reported in Supplementary Table S2 and then loaded on 24-cm-long IPG-strips with a 3-10 NL pH range (GE Healthcare). The first and the second dimensions were carried out as previously described^62^.

#### Analysis of gel images

Gels were scanned using a fluorescent scanner (Typhoon 9400, GE Healthcare). Proteins were visualized at the specific excitation/emission wavelengths for each dye: 532/580 nm for Cy3, 633/670 nm for Cy5 and 488/520 nm for Cy2. Gel images were analyzed using the DeCyder software version 5.02 (GE Healthcare) as previously described^63^. The intensity of each spot was expressed as a mean value of 4 standard abundances calculated for each gel reported in Supplementary Table S2. Spot intensities were then compared in pairs for the three conditions under analysis: CardioLC *versus* control, CardioLC *versus* MMLC, MMLC *versus* control. Only protein spots with at least 1.20-fold change and *p* value ≤ 0.05 were considered significantly altered.

#### *In-gel protein digestion*, *MS analysis and protein identification*

For protein identification, a semipreparative gel was prepared by loading 0.5 mg of unlabeled protein extracts and stained as previously described^64^. Protein spots were picked using an Ettan Spot Picker (GE Healthcare), washed in 50 mM ammonium bicarbonate and 50% acetonitrile and then hydrolyzed with trypsin as described^65,66^. Peptide mixtures were analyzed by liquid chromatography-tandem mass spectrometry (LC-MS/MS) using the LC/MSD Trap XCT Ultra (Agilent Technologies, Palo Alto, CA, USA) equipped with an 1100 HPLC system and a chip cube (Agilent Technologies) as previously described^17^. Mascot software (Matrix Science, London, UK) was used for protein identification against NCBI database (version of February 2015) containing 61,078,976 sequences and using the following standard parameters: *Homo Sapiens*; one missed cleavage; carboxyamidomethylation of Cys, partial Met oxidation and putative modification of Gln to pyro-Glu, mass tolerance of 300 ppm on precursor ions and 0.6 Da on the product ions, individual ion scores > 44.

### Label-free differential analysis

Three biological replicates per condition were analyzed, each one in three technical replicates, making a total of 27 distinct LC-MS/MS runs. Briefly, 15 µg of each sample were reduced (10 mM DTT, 1 h, 37 °C), alkylated (20 mM IAA, 30 min, RT) and digested in-solution (o/n, 37 °C) by sequencing-grade modified trypsin (Promega, Madison, WI, USA) (enzyme:substrate 1:10). After digestion, peptide were cleaned up on a homemade Empore C18 column (3 M, St. Paul, MN, USA)^67^. For each sample, 1 µg was analyzed in a LTQ Velos Pro (Thermo Fisher Scientific, Waltham, MA, USA) coupled to a nano-LC (Proxeon, Odense, Denmark) and peptides were separated by reversed-phase chromatography using a 12-cm column with an inner diameter of 75 μm, packed with 5 μm C18 particles (Nikkyo Technos Co., Ltd. Japan). Chromatographic gradients started at 3% buffer B with a flow rate of 300 nL/min and gradually increased to 7% buffer B in 1 min and to 35% buffer B in 120 min. After each analysis, the column was washed for 10 min with 90% buffer B (Buffer A: 0.1% formic acid in water. Buffer B: 0.1% formic acid in acetonitrile). The mass spectrometer was operated in data-dependent acquisition (DDA) mode, so that each survey scan was followed by the MS/MS of the 10 most intense multiple charged ions, which were selected for fragmentation at normalized collision energy of 35%. Fragment ion spectra produced via collision-induced dissociation (CID) were acquired in the linear ion trap. Raw MS/MS files were processed using Proteome Discoverer version 1.4.1.12 (Thermo Fisher Scientific, Bremen). Peak lists were searched using Mascot software version 2.4.1 against the human SwissProt database (version of July 2013) containing 20,277 protein entries, a list of 598 common contaminants, and all the corresponding decoy entries. The precursor ion mass tolerance was set to 7 ppm, and the fragment ion mass tolerance was set to 0.5 Da. Up to three missed cleavages were allowed, and Oxidation (M) and Acetylation (Protein N-term) were defined as variable modifications, whereas carbamidomethylation (C) was set as fixed modification. The resulting proteins and peptides were filtered using 1% False Discovery Rate. Protein relative abundances were calculated using the R Package MSstats (http://www.msstats.org/); only those peptides that were present in at least three replicates per condition were used for protein quantification. Three pairwise comparisons were performed: i) CardioLC-treated hCFs *versus* untreated hCFs; ii) CardioLC-treated hCFs *versus* MMLC-treated hCFs; and iii) MMLC-treated hCFs *versus* untreated cells. Only proteins with an adjusted *p* value < 0.01, identified with > 2 distinct peptides and appearing in > 75% of all replicates (i.e. in > 21 replicates) were considered.

#### Bioinformatic analysis

Differentially expressed proteins identified with the two proteomic methodologies were analyzed for functional annotation by DAVID Bioinformatic Resource v6.7 (http://www.david.abcc.ncifcrf.gov) and for identification and visualization of molecular networks by Ingenuity Pathway Analysis (IPA) (http://www.ingenuity.com/).

#### Western blot analysis

A subset of proteins from the DIGE and shotgun results (as indicated in the results section) was selected to be validated by western blotting. Selection was based on their fold change, biological role and/or availability of validated antibodies. Each cellular extract (10 μg) was separated on 4–15% polyacrylamide gradient gels (Mini-PROTEAN TGX gels, Biorad, Hercules, CA, USA) under denaturing and reducing conditions; three biological replicates per condition were assessed, each one considered as the average of at least two technical replicates. Proteins were electroblotted onto PVDF membranes using a BioRad Transblot Turbo apparatus. The following primary antibodies were used: rabbit polyclonal anti-CSRP2 (Proteintech, Rosemont, IL, USA) and anti CAND1 (Bethyl Laboratories, Montgomery, TX, USA); mouse monoclonal anti-talin (Merk Millipore, Darmstadt, Germany); anti-HSP27, anti-VDAC1, anti-PSMB2, anti-transgelin and anti-vimentin (all from Santa Cruz Biotechnology, Santa Cruz, CA, USA). Immunoblots were detected by chemiluminescence using Millipore Immobilon HRP substrate. Digital images were acquired with an ImageQuant LAS4000 apparatus, using the ImageQuant LAS4000 control software (GE Healthcare); densitometry measurements were performed and analyzed using Image J software. β-actin (mouse monoclonal antibody, Santa Cruz), tested on the same membrane as each protein under study, was used to estimate the total amount of loaded cellular proteins. In each membrane, the results (expressed as the ratio between the raw densitometry signal of each protein over that of the corresponding β-actin band) were normalized as percentage of the mean of controls. Results were statistically evaluated by Student’s t-test (*p* values < 0.05 were considered significant).

To verify the generalizability of the proteomic results, we assessed the levels of the 8 proteins indicated above by western blot on the protein lysates of hCFs incubated with the LCs listed in Table 1. The latter validation analyses were performed on a batch of commercial hCFs distinct from that used in the proteomic analyses, on two biological replicates, each analyzed in two technical replicates. CardioLC-1 and MMLC-2 were included in the evaluations as internal controls. Differences in mean values among comparison groups were determined by one-way Analysis of Variance (ANOVA). Normal distribution of data was assessed beforehand using Shapiro-Wilk test. Statistical analysis was performed using StatView software; Fisher’s Least Significant Difference (*p* values < 0.05) and Bonferroni correction (*p* value < 0.0167) were used as post-hoc tests.

### Viability, necrosis and apoptosis assays

#### Cell viability

CellTiter 96® AQueous Cell Proliferation Assay (MTS) (Promega, Madison, WI, USA) was used according to the manufacturer’s instruction. Briefly, 20 µl of the CellTiter 96® AQueous One Solution Reagent were added directly into each culture well. After 3 hours of incubation at 37 °C, the absorbance at 492 nm was measured with a standard microplate reader (Infinite® F200, Tecan Group Ltd, Männedorf, Switzerland). In this assay, the quantity of formazan produced is directly proportional to the number of cells alive. Relative cell viability (%) was calculated using control wells containing hCFs grown in standard growth medium (Untreated) as reference condition. Each experiment was performed using five biological replicates, each one in three technical replicates.

#### LDH measurement

CytoTox96® Non-Radioactive Cytotoxicity assay (Promega, Madison, WI, USA) was used according to the manufacturer’s instructions. Briefly, 50 µl of cell culture medium was collected from each well and plated into a new microtiter plate and 50 µl of substrate solution was added. Plates were incubated for 30 minutes at room temperature; then, the reaction was blocked with a stop solution and the absorbance at 492 nm was measured with a standard microplate reader (Infinite® F200, Tecan Group Ltd, Männedorf, Switzerland). Each experiment was performed using five biological replicates, each one in three technical replicates and the amount of LDH released by hCFs was expressed as relative amount related to control wells containing fully lysed hCFs.

#### TUNEL assay

TUNEL was performed with the DeadEnd™ Fluorometric TUNEL System Assay (Promega, Madison, WI, USA) according to the manufacturer’s instructions. Untreated cells were used as negative control. A positive control was also used but it is not reported in the Fig. 5. At the end of the treatment, hCFs were fixed with 4% paraformaldehyde solution for 25 minutes at 4 °C, permeabilized with 0.2% Triton X-100 solution in PBS for 5 minutes and finally incubated with 50 µl TdT reaction mix for 60 minutes at 37 °C in a humidified chamber. At the end of incubation, the reaction was blocked with a stop solution and the nuclei were counterstained with Hoechst 33258 (SIGMA-ALDRICH, St. Louis, MO, USA). Slides were mounted with Vectashield mounting medium (Vector Laboratories Inc., Burlingame, CA, USA) and TUNEL-positive cells and the total cell number per view were counted with a Zeiss Axio Observer Z1 microscope (Carl Zeiss, Milan, Italy) equipped with the Apotome system.

#### Measurement of Intracellular ROS

After exposure to LCs, hCFs were incubated for 30 minutes with 25 µM redox-sensitive fluorophore, dichlorofluorescein-diacetate (DCF-DA) in the Image-iT™ LIVE Green Reactive Oxygen Species Detection Kit for microscopy (Life Technologies Corporation, Carlsbad, CA, USA). DCF fluorescence was visualized using a Zeiss Axio Observer Z1 microscope (Carl Zeiss, Milan, Italy) equipped with the Apotome system. The quantification of fluorescence signals was performed with Axio Vision 4.8.2 Software (Carl Zeiss, Milan, Italy). The production of ROS after LC exposure relative to the production of ROS in the untreated cells was calculated.

#### Statistical analysis of cell-based assays

All cellular assays were performed using five biological replicates, each one in three technical replicates. All results are presented as the mean plus or minus standard deviation. Differences in mean values among comparison groups were determined by ANOVA after assumptions of normal distribution and homogeneity of variances were verified. Differences were considered statistically significantly when *p* values < 0.05. Statistical analysis was performed with the InStat^TM^ software.

## Electronic supplementary material

Supplementary Information

## References

[1] Sipe JD (2016). Amyloid fibril proteins and amyloidosis: chemical identification and clinical classification International Society of Amyloidosis 2016 Nomenclature Guidelines. Amyloid.

[2] Merlini G, Wechalekar AD, Palladini G (2013). Systemic light chain amyloidosis: an update for treating physicians. Blood.

[3] Merlini G, Palladini G (2013). Light chain amyloidosis: the heart of the problem. Haematologica.

[4] Sapp V, Jain M, Liao R (2016). Viewing extrinsic proteotoxic stress through the lens of amyloid cardiomyopathy. Physiology (Bethesda).

[5] Lavatelli F, Albertini R, Di Fonzo A, Palladini G, Merlini G (2014). Biochemical markers in early diagnosis and management of systemic amyloidoses. Clin Chem Lab Med.

[6] Merlini G (2016). Rationale, application, and clinical qualification for NT-proBNP as a surrogate end point in pivotal clinical trials in patients with AL amyloidosis. Leukemia.

[7] Palladini G (2006). Circulating amyloidogenic free light chains and serum N-terminal natriuretic peptide type B decrease simultaneously in association with improvement of survival in AL. Blood.

[8] Palladini G (2012). New criteria for response to treatment in immunoglobulin light chain amyloidosis based on free light chain measurement and cardiac biomarkers: impact on survival outcomes. J Clin Oncol.

[9] Guan J (2014). Lysosomal dysfunction and impaired autophagy underlie the pathogenesis of amyloidogenic light chain-mediated cardiotoxicity. EMBO Mol Med.

[10] Guan J (2013). Stanniocalcin1 is a key mediator of amyloidogenic light chain induced cardiotoxicity. Basic Res Cardiol.

[11] Mishra S (2013). Human amyloidogenic light chain proteins result in cardiac dysfunction, cell death, and early mortality in zebrafish. Am J Physiol Heart Circ Physiol.

[12] Shi J (2010). Amyloidogenic light chains induce cardiomyocyte contractile dysfunction and apoptosis via a non-canonical p38alpha MAPK pathway. Proc Natl Acad Sci USA.

[13] Brenner DA (2004). Human amyloidogenic light chains directly impair cardiomyocyte function through an increase in cellular oxidant stress. Circ Res.

[14] Diomede L (2014). A Caenorhabditis elegans-based assay recognizes immunoglobulin light chains causing heart amyloidosis. Blood.

[15] Monis GF (2006). Role of endocytic inhibitory drugs on internalization of amyloidogenic light chains by cardiac fibroblasts. Am J Pathol.

[16] Trinkaus-Randall V (2005). Cellular response of cardiac fibroblasts to amyloidogenic light chains. Am J Pathol.

[17] Lavatelli F (2015). Novel mitochondrial protein interactors of immunoglobulin light chains causing heart amyloidosis. FASEB J.

[18] Liao R (2001). Infusion of light chains from patients with cardiac amyloidosis causes diastolic dysfunction in isolated mouse hearts. Circulation.

[19] Marin-Argany M (2016). Cell Damage in Light Chain Amyloidosis: fibril internalization, toxicity and cell-mediated seeding. J Biol Chem.

[20] Porter KE, Turner NA (2009). Cardiac fibroblasts: at the heart of myocardial remodeling. Pharmacol Ther.

[21] Jugdutt BI (2003). Ventricular remodeling after infarction and the extracellular collagen matrix: when is enough enough?. Circulation.

[22] Sikkink LA, Ramirez-Alvarado M (2010). Cytotoxicity of amyloidogenic immunoglobulin light chains in cell culture. Cell Death Dis.

[23] Megger DA (2013). Proteomic differences between hepatocellular carcinoma and nontumorous liver tissue investigated by a combined gel-based and label-free quantitative proteomics study. Mol Cell Proteomics.

[24] Migrino RQ (2010). Systemic and microvascular oxidative stress induced by light chain amyloidosis. Int J Cardiol.

[25] Diomede, L. *et al*. Cardiac light chain amyloidosis: The role of metal ions in oxidative stress and mitochondrial damage. *Antioxid Redox Signal*, 10.1089/ars.2016.6848 (2017). 10.1089/ars.2016.6848 PMC5567464 28132512

[26] Yao Q, Qu X, Yang Q, Wei M, Kong B (2009). CLIC4 mediates TGF-beta1-induced fibroblast-to-myofibroblast transdifferentiation in ovarian cancer. Oncol Rep.

[27] Hegde ML (2012). Enhancement of NEIL1 protein-initiated oxidized DNA base excision repair by heterogeneous nuclear ribonucleoprotein U (hnRNP-U) via direct interaction. J Biol Chem.

[28] Singh S (2013). Aldehyde dehydrogenases in cellular responses to oxidative/electrophilic stress. Free Radic Biol Med.

[29] Swomley AM (2014). Abeta, oxidative stress in Alzheimer disease: evidence based on proteomics studies. Biochim Biophys Acta.

[30] Baek HY (2004). Oxidative-stress-related proteome changes in Helicobacter pylori-infected human gastric mucosa. Biochem J.

[31] Ortuño-Sahagún D, Pallàs M, Rojas-Mayorquín AE (2014). Oxidative stress in aging: advances in proteomic approaches. Oxid Med Cell Longev.

[32] Baraibar MA (2011). Oxidative stress-induced proteome alterations target different cellular pathways in human myoblasts. Free Radic Biol Med.

[33] Arntzen MØ, Thiede B (2012). ApoptoProteomics, an integrated database for analysis of proteomics data obtained from apoptotic cells. Mol Cell Proteomics.

[34] Brambilla F (2013). Shotgun protein profile of human adipose tissue and its changes in relation to systemic amyloidoses. J Proteome Res.

[35] Albigès-Rizo C, Frachet P, Block MR (1995). Down regulation of talin alters cell adhesion and the processing of the alpha 5 beta 1 integrin. J Cell Sci.

[36] Bogatan S (2015). Talin is required continuously for cardiomyocyte remodeling during heart growth in Drosophila. PLoS One.

[37] Schevzov G, Lloyd C, Gunning P (1995). Impact of altered actin gene expression on vinculin, talin, cell spreading, and motility. DNA Cell Biol.

[38] Varisli L (2011). Ubiquitously expressed hematological and neurological expressed 1 downregulates Akt-mediated GSK3β signaling, and its knockdown results in deregulated G2/M transition in prostate cells. DNA Cell Biol.

[39] Agocha A, Sigel AV, Eghbali-Webb M (1997). Characterization of adult human heart fibroblasts in culture: a comparative study of growth, proliferation and collagen production in human and rabbit cardiac fibroblasts and their response to transforming growth factor-beta1. Cell Tissue Res.

[40] Rohr S (2011). Cardiac fibroblasts in cell culture systems: myofibroblasts all along?. J Cardiovasc Pharmacol.

[41] Lavatelli F (2008). Amyloidogenic and associated proteins in systemic amyloidosis proteome of adipose tissue. Mol Cell Proteomics.

[42] Cuanalo-Contreras K, Mukherjee A, Soto C (2013). Role of protein misfolding and proteostasis deficiency in protein misfolding diseases and aging. Int J Cell Biol.

[43] Riederer BM, Leuba G, Vernay A, Riederer IM (2011). The role of the ubiquitin proteasome system in Alzheimer’s disease. Exp Biol Med (Maywood).

[44] Moreira PI, Carvalho C, Zhu X, Smith MA, Perry G (2010). Mitochondrial dysfunction is a trigger of Alzheimer’s disease pathophysiology. Biochim Biophys Acta.

[45] Kumar A, Singh A (2015). A review on mitochondrial restorative mechanism of antioxidants in Alzheimer’s disease and other neurological conditions. Front Pharmacol.

[46] Pagani L, Eckert A (2011). Amyloid-Beta interaction with mitochondria. Int J Alzheimers Dis.

[47] Harner ME (2014). Aim24 and MICOS modulate respiratory function, tafazzin-related cardiolipin modification and mitochondrial architecture. Elife.

[48] Harner M (2011). The mitochondrial contact site complex, a determinant of mitochondrial architecture. EMBO J.

[49] Robledo S (2008). The role of human ribosomal proteins in the maturation of rRNA and ribosome production. RNA.

[50] Wang W (2015). Ribosomal proteins and human diseases: pathogenesis, molecular mechanisms, and therapeutic implications. Med Res Rev.

[51] Armistead J, Triggs-Raine B (2014). Diverse diseases from a ubiquitous process: the ribosomopathy paradox. FEBS Lett.

[52] Ding Q, Markesbery WR, Chen Q, Li F, Keller JN (2005). Ribosome dysfunction is an early event in Alzheimer’s disease. J Neurosci.

[53] Sherman MY, Qian SB (2013). Less is more: improving proteostasis by translation slow down. Trends Biochem Sci.

[54] Assinder SJ, Stanton JA, Prasad PD (2009). Transgelin: an actin-binding protein and tumour suppressor. Int J Biochem Cell Biol.

[55] Tanaka K (2013). Circulating matrix metalloproteinases and tissue inhibitors of metalloproteinases in cardiac amyloidosis. J Am Heart Assoc.

[56] Backstrom JR, Lim GP, Cullen MJ, Tökés ZA (1996). Matrix metalloproteinase-9 (MMP-9) is synthesized in neurons of the human hippocampus and is capable of degrading the amyloid-beta peptide (1–40). J Neurosci.

[57] Willis MS, Patterson C (2013). Proteotoxicity and cardiac dysfunction–Alzheimer’s disease of the heart?. N Engl J Med.

[58] Rognoni P (2013). A Strategy for Synthesis of Pathogenic Human Immunoglobulin Free Light Chains in E. coli. PLoS One.

[59] Gertz MA (2005). Definition of organ involvement and treatment response in immunoglobulin light chain amyloidosis (AL): a consensus opinion from the 10th International Symposium on Amyloid and Amyloidosis, Tours, France, 18–22 April 2004. Am J Hematol.

[60] Pan C, Gnad F, Olsen JV, Mann M (2008). Quantitative phosphoproteome analysis of a mouse liver cell line reveals specificity of phosphatase inhibitors. Proteomics.

[61] Imperlini E, Orrù S, Corbo C, Daniele A, Salvatore F (2014). Altered brain protein expression profiles are associated with molecular neurological dysfunction in the PKU mouse model. J Neurochem.

[62] Imperlini E (2015). Synergistic effect of DHT and IGF-1 hyperstimulation in human peripheral blood lymphocytes. Proteomics.

[63] Caterino M (2013). Differential proteomic analysis in human cells subjected to ribosomal stress. Proteomics.

[64] Caterino M (2015). The proteome of cblC defect: *in vivo* elucidation of altered cellular pathways in humans. J Inherit Metab Dis.

[65] Nigro E (2015). Differentially expressed and activated proteins associated with non small cell lung cancer tissues. Respir Res.

[66] Spaziani S (2014). Insulin-like growth factor 1 receptor signaling induced by supraphysiological doses of IGF-1 in human peripheral blood lymphocytes. Proteomics.

[67] Rappsilber J, Mann M, Ishihama Y (2007). Protocol for micro-purification, enrichment, pre-fractionation and storage of peptides for proteomics using StageTips. Nat Protoc.

