# Supplementary material for: Proteotoxicity in cardiac amyloidosis: amyloidogenic light chains affect the levels of intracellular proteins in human heart cells
Source: Sci Rep. 2017 Nov 15;7:15661. doi: 10.1038/s41598-017-15424-3 (PMC5688098; doi:10.1038/s41598-017-15424-3)
Supplement: Supplementary file 1 — Supplementary Information [file 41598_2017_15424_MOESM1_ESM.pdf]

Proteotoxicity in cardiac amyloidosis: amyloidogenic light chains affect the levels of intracellular proteins in human heart cells

Esther Imperlini[1,2], Massimiliano Gnecchi[3,4,5], Paola Rognoni[6], Eduard Sabidò[7,8], Maria Chiara Ciuffreda[3], Giovanni Palladini[6], Guadalupe Espadas[7,8], Francesco Mattia Mancuso[7,8], Margherita Bozzola[6], Giuseppe Malpasso[3], Veronica Valentini[6], Giuseppina Palladini[9], Stefania Orrù[1,2,10], Giovanni Ferraro[6], Paolo Milani[6], Stefano Perlini[9], Francesco Salvatore[2,11,*], Giampaolo Merlini[6,*], Francesca Lavatelli[6]

[1]IRCCS SDN, Naples, Italy

[2]CEINGE–Biotecnologie Avanzate, Naples, Italy

[3] Coronary Care Unit and Laboratory of Experimental Cardiology for Cell and Molecular Therapy, Fondazione IRCCS Policlinico San Matteo, Pavia, Italy.

[4] Department of Molecular Medicine, Unit of Cardiology, University of Pavia, Pavia, Italy.

[5] Department of Medicine, University of Cape Town, Cape Town, South Africa

[6]Amyloidosis Research and Treatment Center, Department of Molecular Medicine, Fondazione IRCCS Policlinico San Matteo and University of Pavia, Italy

[7]Centre for Genomic Regulation (CRG), The Barcelona Institute for Science and Technology, Barcelona, Spain

[8]Universitat Pompeu Fabra (UPF), Barcelona, Spain

[9]Department of Internal Medicine, Fondazione IRCCS Policlinico San Matteo and University of Pavia, Italy

[10]Department of Movement Sciences, "Parthenope" University, Naples, Italy

[11]Department of Molecular Medicine and Medical Biotechnologies, University of Naples "Federico II", Italy

***Correspondence and requests for materials should be addressed to F.S. (e-mail: salvator@unina.it) and G.M. (e-mail: gmerlini@unipv.it)

Supplementary Tables

Table S1. Proteins identified as specific component of the proteome of CardioLC-treated cells by shotgun analysis, in comparison with control cells.

**CardioLC *versus* control cells**				
**Gene name**	**Protein name**	**UniProt**	**Control hCF**	**CardioLC**
IGLL5	Immunoglobulin lambda-like polypeptide 5	B9A064	absent	**present**
ICAM1	Intercellular adhesion molecule 1	P05362	absent	**present**
BPGM	Bisphosphoglycerate mutase	P07738	absent	**present**
IGLC2	Ig lambda-2 chain C regions	P0CG05	absent	**present**
VCAN	Versican core protein	P13611	absent	**present**
SNRPB	Small nuclear ribonucleoprotein-associated proteins B and B'	P14678	absent	**present**
M6PR	Cation-dependent mannose-6-phosphate receptor	P20645	**present**	absent

Table S2. 2D DIGE Experimental Design.

Gel	Cy3 (50 µg of protein lysate)	Cy5 (50 µg of protein lysate)	Cy2 (50 µg of protein lysate)
1	Control replicate 1	CardioLC replicate 3	Pooled standard
2	Control replicate 2	CardioLC replicate 4	Pooled standard
3	CardioLC replicate 1	MMLC replicate 3	Pooled standard
4	CardioLC replicate 2	MMLC replicate 4	Pooled standard
5	MMLC replicate 1	Control replicate 3	Pooled standard
6	MMLC replicate 2	Control replicate 4	Pooled standard

Table S3. Proteins differentially represented both in hCF treated with CardioLC and in adipose tissue of patients affected by AL amyloidosis.

Gene	Protein	Localization	Direction	Comparison*
TLN1	Talin-1	CT	↓	1
FLNA	Filamin-A	CT	↓	1
ANXA5	Annexin A5	CY	↓	1
SERPINH1	Serpin H1	ER	↓	2
AHNAK	AHNAK protein	N	↓	1
ATP5A1	ATP synthase subunit alpha	M	↓	2
EHD2	EH domain-containing protein 2	CM	↓	2
CLTC	Clathrin heavy chain 1	CM	↓	1
MYH9	Myosin-9	CT	↑	2
LMNA	Prelamin-A/C	N	↓	3
FN1	Fibronectin	ECM	≠ (↑ in tissue; ↓in hCF)	1
VCL	Vinculin	CT	≠(↑ in tissue; ↓in hCF)	2
VIM	Vimentin	CT	≠ (↑ in hCF; ↓in tissue)	1
HADHB	Trifunctional enzyme subunit beta, mitochondrial	M	≠ (↑ in tissue; ↓in hCF) **	2
COL1A2	Collagen alpha-2(I) chain	ECM	≠ (↑ in tissue; ↓in hCF)	1

* Comparison 1: CardioLC vs Control and vs MMLC; Comparison 2: CardioLC vs Control only; Comparison 3: CardioLC versus MMLC only
** HADHA subunit in fat tissue
CY: cytoplasm; ER: endoplasmic reticulum; N: nucleus; M: mitochondrion; CM: cell membrane; ECM: extracellular matrix; CT: cytoskeleton.

Supplementary Figures

a

CardioLC-treated cells *versus* control cells
Cancer, Cell Death and Survival, Organismal Injury and Abnormalities

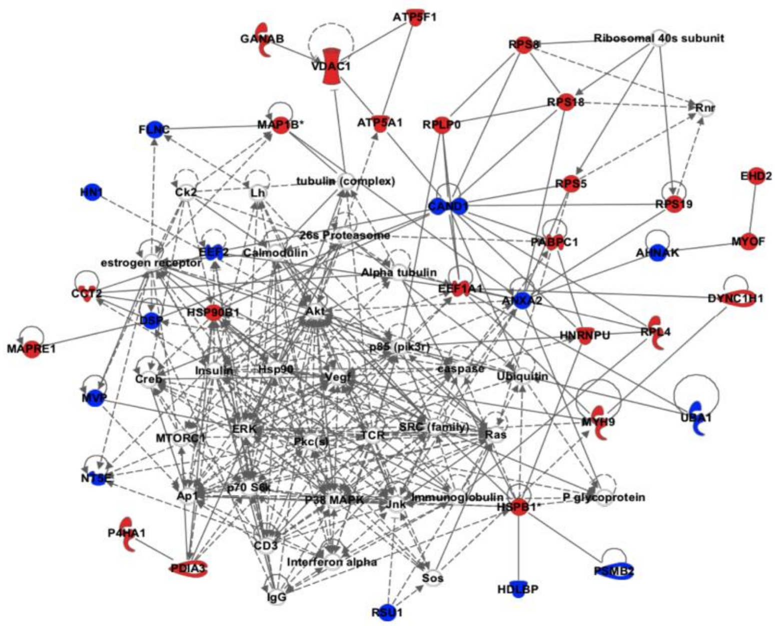

b

CardioLC-treated cells *versus* control cells
Cell Morphology, Cellular Assembly and Organization, Cellular Function and Maintenance

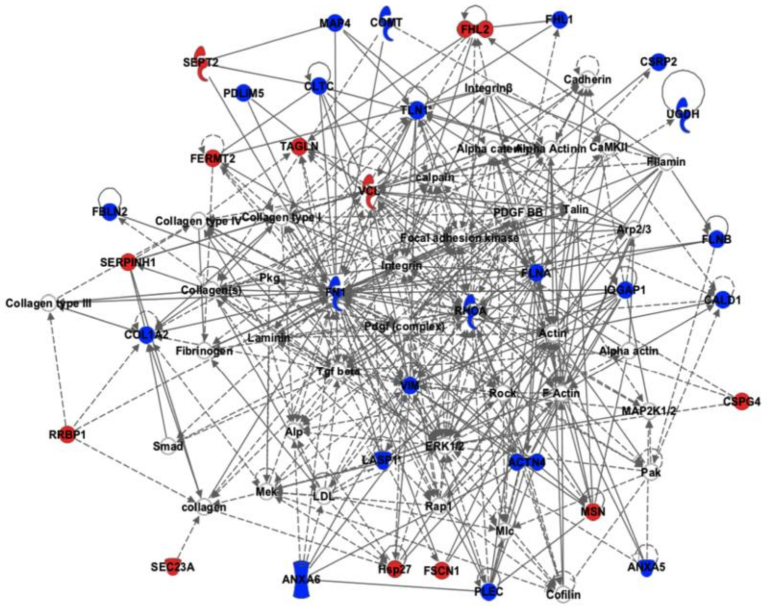

c

CardioLC-treated cells *versus* MMLC-treated cells
Developmental Disorder, Hereditary Disorder, Organismal Injury and Abnormalities

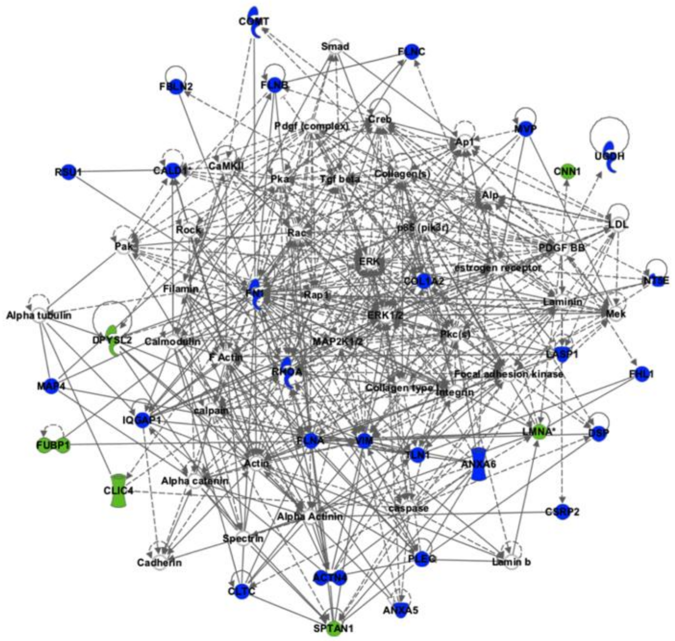

Figure S1. Multidirectional interaction networks according to IPA software. (**a**) and (**b**) Proteins identified as differentially represented in the CardioLC *vs* control comparison, by taking into account the results of both the shotgun and the 2D DIGE analysis. Red: proteins identified as differential only in the CardioLC *vs* control comparison; blue: proteins identified as differential both in the CardioLC *vs* control and in the CardioLC *vs* MMLC comparison. The differential proteins belong to two high-score biological networks, associated to (**a**) "Cancer, Cell Death and Survival, Organismal Injury and Abnormalities" (score=78) and (**b**) "Cell Morphology, Cellular Assembly and Organization, Cellular Function and Maintenance" (score=67). (**c**) Proteins identified as differential in the CardioLC *vs* MMLC comparison. The differential proteins belong to one high-score biological network associated to (**c**) "Developmental Disorder, Hereditary Disorder, Organismal Injury and Abnormalities. Green: proteins identified as differential only in the CardioLC *vs* MMLC comparison; blue: proteins identified as differential both in the CardioLC *vs* control and in the CardioLC *vs* MMLC comparison.

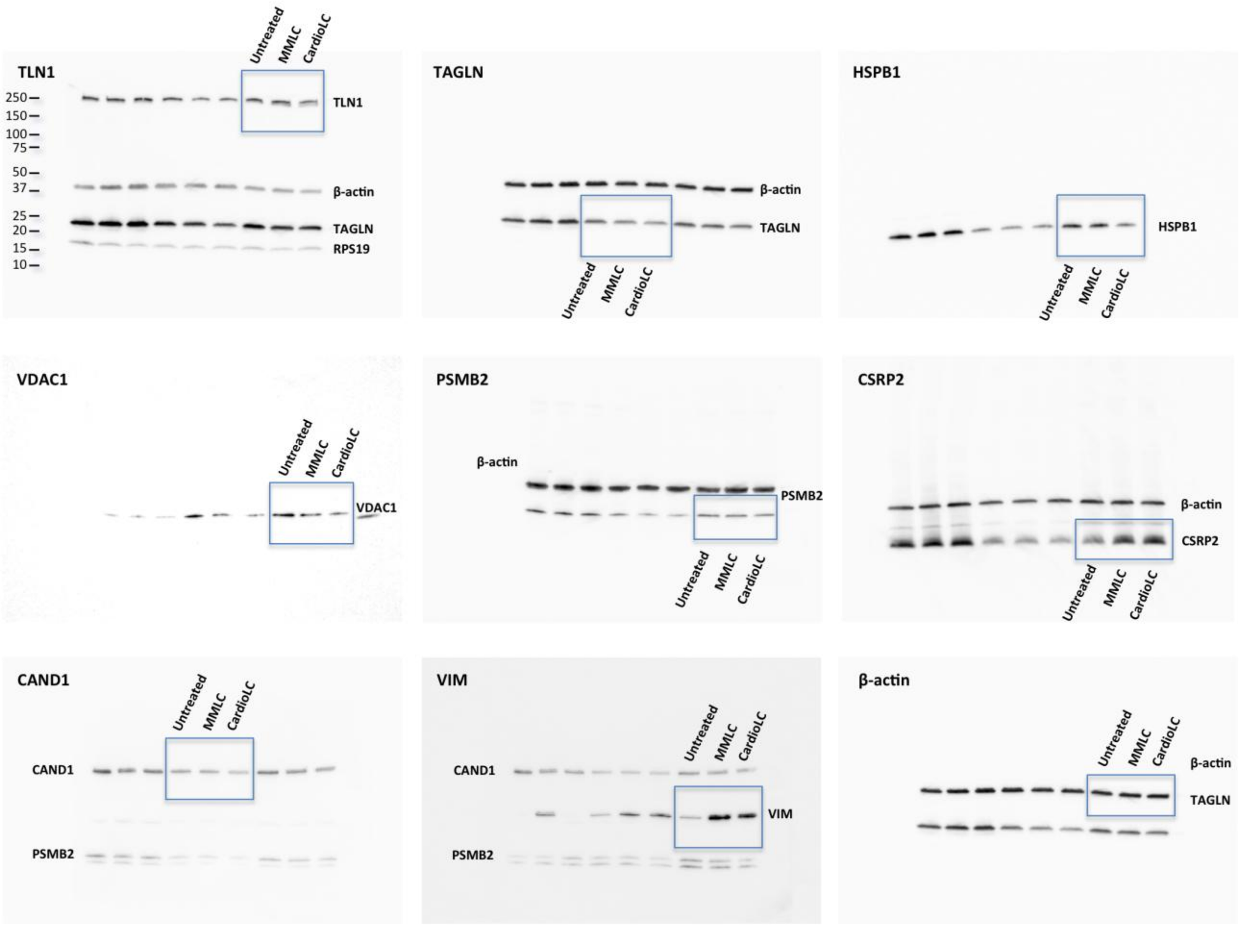

Figure S2. Full length digital images of the western blots displayed in Fig 4. Each image refers to the replicate displayed in the text (boxed) for each indicated protein. The appearance of multiple bands in some images is due to the sequential evaluation of multiple proteins on the same membrane.

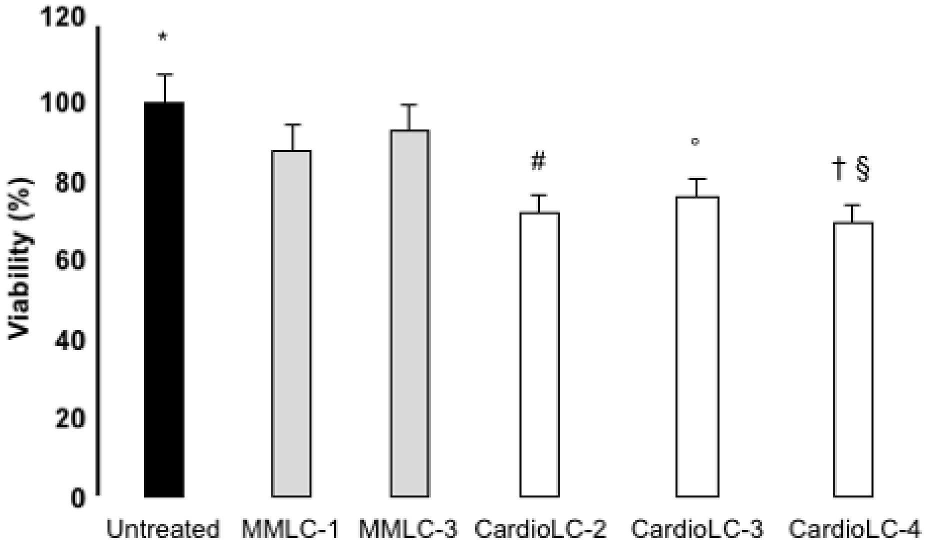

Figure S3. Evaluation of effects on cell viability by different LCs. Cell viability was significantly reduced in hCFs exposed for 24 h to 3 different CardioLCs, compared to hCFs exposed to 2 different MMLC and to untreated ones. **p* value <0.001 *versus* CardioLC-2, CardioLC-3, CardioLC-4; #*p* value <0.01 *versus* MMLC-3; °*p* value <0.05 *versus* MMLC-3; †*p* value <0.01 *versus* MMLC-1; §*p* value <0.001 *versus* MMLC-3.

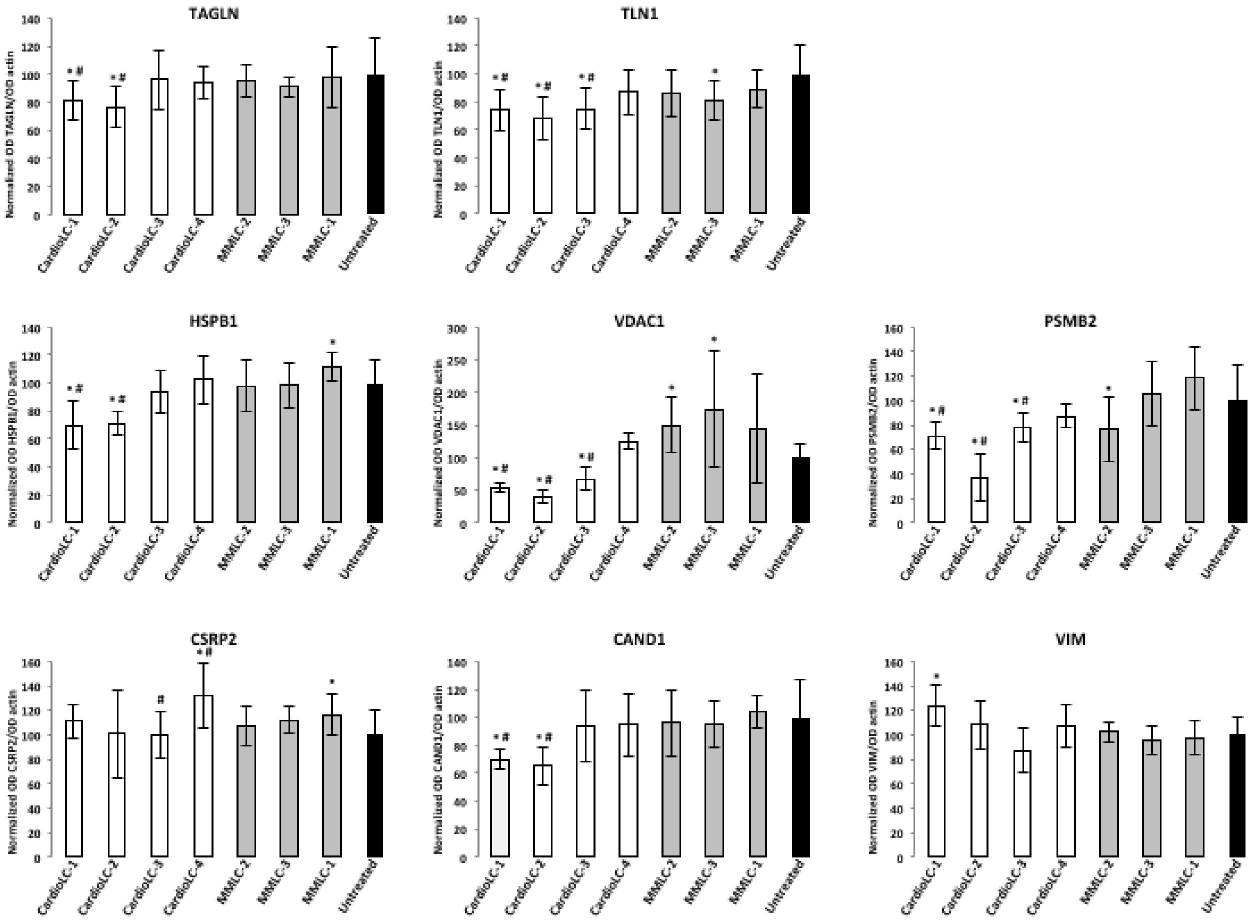

Figure S4. Verification of differentially represented proteins in hCFs incubated with other LCs using western blot. Data referred to each LC are individually presented. The graphs display the ratio (average values; bars represent standard deviations) between the signal of each protein and the corresponding β-actin, normalized against the average of the corresponding control cells. Results were statistically evaluated by 2-tailed unpaired Student's t-test. **p* value < 0.05 *versus* Untreated; #*p* value < 0.05 *versus* MMLC.
